# Addressing widespread misidentifications of traditional medicinal mushrooms in *Sanghuangporus* (*Basidiomycota*) through ITS barcoding and designation of reference sequences

**DOI:** 10.1186/s43008-021-00059-x

**Published:** 2021-04-15

**Authors:** Shan Shen, Shi-Liang Liu, Ji-Hang Jiang, Li-Wei Zhou

**Affiliations:** 1 State Key Laboratory of Mycology, Institute of Microbiology, Chinese Academy of Sciences, Beijing, 100101 China; 2 University of Chinese Academy of Sciences, Beijing, 100049 China; 3 Institute of Applied Ecology, Chinese Academy of Sciences, Shenyang, 110016 China

**Keywords:** *Hymenochaetaceae*, Phylogeny, Species boundary, Taxonomy, Wood-inhabiting fungi, One new taxon

## Abstract

“Sanghuang” refers to a group of important traditionally-used medicinal mushrooms belonging to the genus *Sanghuangporus*. In practice, species of *Sanghuangporus* referred to in medicinal studies and industry are now differentiated mainly by a BLAST search of GenBank with the ITS barcoding region as a query. However, inappropriately labeled ITS sequences of “Sanghuang” in GenBank restrict accurate species identification and, to some extent, the utilization of these species as medicinal resources. We examined all available 271 ITS sequences related to “Sanghuang” in GenBank including 31 newly submitted sequences from this study. Of these sequences, more than half were mislabeled so we have now corrected the corresponding species names. The mislabeled sequences mainly came from strains utilized by non-taxonomists. Based on the analyses of ITS sequences submitted by taxonomists as well as morphological characters, we separate the newly described *Sanghuangporus subbaumii* from *S. baumii* and treat *S. toxicodendri* as a later synonym of *S. quercicola*. Fourteen species of *Sanghuangporus* are accepted, with intraspecific distances up to 1.30% (except in *S. vaninii*, *S. weirianus* and *S. zonatus*) and interspecific distances above 1.30% (except between *S. alpinus* and *S. lonicerinus*, and *S. baumii* and *S. subbaumii*). To stabilize the concept of these 14 species of *Sanghuangporus*, their taxonomic information and reliable ITS reference sequences are provided. Moreover, ten potential diagnostic sequences are provided for Hyperbranched Rolling Circle Amplification to rapidly confirm three common commercial species, viz. *S. baumii*, *S. sanghuang*, and *S. vaninii*. Our results provide a practical method for ITS barcoding-based species identification of *Sanghuangporus* and will promote medicinal studies and commercial development from taxonomically correct material.

**Supplementary Information:**

The online version contains supplementary material available at 10.1186/s43008-021-00059-x.

## INTRODUCTION

Many macrofungi are established in traditional medicine and possess diverse properties (Wu et al. 2019a). “Sanghuang” comprises an important group of wood-inhabiting mushrooms that have been utilized in traditional medicine in China and adjacent countries for 2000 years (Zhou et al. 2020). Modern scientific studies have revealed several medicinal attributes of “Sanghuang”, including antitumor, antioxidant, anti-inflammation, and immunomodulation activities (Zhou et al. 2020). This fungal resource has also attracted the attentions of fungal chemists and pharmacologists outside Asia (Chepkirui et al. 2018; Cheng et al. 2019). Natural products, such as polysaccharides, polyphenols, pyrones and terpenes are the bioactive compounds responsible for the medicinal properties of “Sanghuang” (Zhou et al. 2020). Today, “Sanghuang” is mainly consumed in a brewed tea made from small pieces of cultivated basidiomes or occasionally powdered mycelia.

Like other wood-inhabiting traditional medicinal mushrooms, such as “Lingzhi” (Cao et al. 2012; Wang et al. 2012; Yao et al. 2013, 2020; Dai et al. 2017), “Niuchangchih” (Wu et al. 2012b, 2012c) and “Fuhling” (Redhead and Ginns 2006), there has been much debate about the taxonomic identity of “Sanghuang”. Most fungal taxonomists now agree that “Sanghuang” is represented by species of *Sanghuangporus* (Zhou et al. 2020). Fourteen species have been described and accepted as members of *Sanghuangporus*: 11 species in Asia, and one in each of Africa, Europe, and North America (Zhou et al. 2020). In addition, more new species await to be described from Africa (Chepkirui et al. 2018; Cheng et al. 2019) and perhaps other parts of the world. Besides morphological and ecological (host preference) characters, the ITS barcoding region provides the most powerful tool for differentiating species of the genus. For example, more than half of the known species of *Sanghuangporus* were discovered with the aid of the ITS region alone (Wu et al. 2012a, 2019b; Tian et al. 2013; Ghobad-Nejhad 2015; Tomšovský 2015; Zhu et al. 2017). Moreover, the reliability of the ITS region for species differentiation in the genus has been substantiated by a multilocus-based phylogenetic analysis (Zhu et al. 2019). Consequently, Zhou et al. (2020) reported ITS sequences from reliably identified voucher collections of the known species in the genus.

Transdisciplinary studies on *Sanghuangporus* have been performed to promote the utilization of this medicinal resource (Zhou et al. 2016; Cai et al. 2019; Zhu et al. 2019; Shao et al. 2020). Most of these studies aimed to identify their materials via a BLAST search of GenBank (https://www.ncbi.nlm.nih.gov/genbank/) using the ITS barcoding region as the query. However, even though each of the 14 species of *Sanghuangporus* has a reliable ITS sequence accession number (Zhou et al. 2020), it is not always easy to determine material in hand by a simple ITS-based BLAST search. This is a consequence of redundant and even incorrectly labeled ITS sequences in GenBank (Nilsson et al. 2006; Hofstetter et al. 2019). With inaccurately identified sequences emerging as potential matches, more collections will inevitably be inaccurately identified and the ITS sequences generated from the inaccurately identified collections will be submitted to GenBank compounding the issue and presenting new obstacles for later accurate identification. This means that there is high likelihood of medicinal and other attributes being attributed to incorrectly named species of “Sanghuang”. Meanwhile, before the erection of the genus *Sanghuangporus* (Zhou et al. 2016), ITS sequences generated from “Sanghuang” were labeled under other generic names, such as *Inonotus* and *Phellinus*, even though with the correct epithets. This phenomenon confuses researchers who lack taxonomic knowledge, and results in a misapplication of species names to medicinal properties, which then has a negative effect on obtaining permissions from regulatory authorities for commercial development (Zhou 2020).

As stated by Zhou (2020), the use of correct scientific names for fungal species is crucial to studies of traditional Chinese medicine and their commercial exploitation. To facilitate the rational medicinal utilization of *Sanghuangporus*, all ITS sequences related to “Sanghuang” in GenBank should be re-examined to assist species identification. The aim of the current study is therefore to assess the utility of the ITS region for species discrimination in *Sanghuangporus*, and reset the species circumscriptions on the basis of the ITS barcoding region, in order to facilitate the correction of previously mislabeled ITS sequences in GenBank, and to provide candidate diagnostic ITS sequences for use in rapid species identification of *Sanghuangporus* using Hyperbranched Rolling Circle Amplification (HRCA).

## MATERIALS AND METHODS

### Morphological examination

The newly sequenced specimens and strains are deposited in HMAS, IFP and BJFC. The specimens were observed with an Olympus BX43 light microscope (Tokyo, Japan) at magnifications up to 1000×. Microscopic procedure followed Zhou et al. (2016). Specimen sections were prepared in Cotton blue (CB), Melzer’s reagent (IKI), and 5% potassium hydroxide (KOH). All measurements were made from material mounted in heated CB. When presenting the variation of basidiospore sizes, 5% of the measurements were excluded from each end of the range and are given in parentheses. Drawings were made with the aid of a drawing tube. In the text, L = mean basidiospore length (arithmetic average of all measured basidiospores), W = mean basidiospore width (arithmetic average of all measured basidiospores), Q = variation in the L/W ratios between the studied specimens, and (a/b) = number of basidiospores (a) measured from given number (b) of specimens.

### Molecular sequencing

A small piece of the basidiome or culture was taken for DNA extraction, which was performed using a CTAB rapid plant genome extraction kit-DN14 (Aidlab Biotechnologies, Beijing). The crude DNA was used as templates for the PCR amplifications of the ITS region. The primer pairs ITS1F/ITS4 and ITS5/ITS4 (White et al. 1990; Gardes and Bruns 1993) were selected for amplification and subsequent sequencing at the Beijing Genomics Institute. The PCR procedure was as follows: initial denaturation at 95 °C for 3 min, followed by 34 cycles at 94 °C for 40 s, 57.2 °C for 45 s and 72 °C for 1 min, and a final extension at 72 °C for 10 min. All newly generated sequences are deposited in GenBank (Table 1).

**Table 1:** Information of analyzed ITS sequences of *Sanghuangporus*

No.	Species name accepted here	Species name in GenBank	Voucher No.	GenBank No.	Host plant	Geographic origin	Type of material	Identifier of material
1.	*S. alpinus*	*I. alpinus*	Cui 9646	JQ860313^a^	Angiosperm	Tibet, China	Specimen	Tian XM et al.
2.		*I. alpinus*	Cui 9652	JQ860309^a^	Angiosperm	Tibet, China	Specimen	Tian XM et al.
3.		*I. alpinus*	Cui 9658	JQ860310^a^	Angiosperm	Tibet, China	Specimen	Tian XM et al.
4.		*I. alpinus*	Cui 9666	JQ860311^a^	Angiosperm	Tibet, China	Specimen	Tian XM et al.
5.		*S. alpinus*	Cui 12444	MF772782^a^	*Lonicera*	Sichuan, China	Specimen	Zhu L & Cui BK
6.		*S. alpinus*	Cui 12474	MF772783^a^	*Lonicera*	Sichuan, China	Specimen	Zhu L & Cui BK
7.		*S. alpinus*	Cui 12485	MF772781^a^	*Lonicera*	Sichuan, China	Specimen	Zhu L & Cui BK
8.		*I. alpinus*	Yu 35	JQ860312^a^	*Lonicera*	Tibet, China	Specimen	Tian XM et al.
9.		***S. alpinus***	**Yuan 6396 (IFP)**	**MT348577**^a^	***Lonicera***	**Qinghai, China**	**Specimen**	**This study**
10.		***S. alpinus***	**Yuan 6405 (IFP)**	**MT348578**^a^	***Lonicera***	**Qinghai, China**	**Specimen**	**This study**
11.		***S. alpinus***	**Yuan 6438 (IFP)**	**MT343579**^a^	**Angiosperm**	**Qinghai, China**	**Specimen**	**This study**
12.	*S. baumii*	*T. linteus*	ASI 26030	KT862142		South Korea	Strain	Han JG et al.
13.		*T. linteus*	ASI 26086	KT862157		Samchoek, South Korea	Strain	Han JG et al.
14.		*T. linteus*	ASI 26087	KT862158		Mokpo, South Korea	Strain	Han JG et al.
15.		*S. baumii*	ASI 26108	KT862162		Inje, South Korea	Strain	Han JG et al.
16.		*I. baumii*	Cui 3573	JQ860307^a^	*Syringa*	Jilin, China	Specimen	Tian XM et al.
17.		*S. baumii*	Cui 11769	MF772784^a^	Angiosperm	Heilongjiang, China	Specimen	Zhu L & Cui BK
18.		*S. baumii*	Cui 11903	KY328305^a^	*Alnus*	Heilongjiang, China	Specimen	Zhu L & Cui BK
19.		*P. baumii*	Dai 2340	AF534069			Strain	Lim YW et al.
20.		*I. baumii*	Dai 3683	JN642567^a^	*Syringa*	Heilongjiang, China	Strain	Wu SH et al.
21.		*I. baumii*	Dai 3684	JN642568^a^	*Syringa*	Heilongjiang, China	Strain	Wu SH et al.
22.		*I. baumii*	Dai 3694	JN642569^a^	*Syringa*	Heilongjiang, China	Strain	Wu SH et al.
23.		*S. baumii*	Dai 16900	MF772785^a^	*Syringa*	Heilongjiang, China	Specimen	Zhu L & Cui BK
24.		*I. baumii*	FS 656165	HM584807			Strain	Yu TW
25.		*I. baumii*	FS 656164	GU903007			Strain	Yu TW
26.		*I. baumii*	HLJU	KC312696			Strain	Liu Y et al.
27.		*S. baumii*	KUC 10644	MH168100			Strain	Heo YM et al.
28.		*I. baumii*	KUC 20130809–20	KJ668511		South Korea	Specimen	Jang Y & Kim JJ
29.		*I. baumii*	MDJCBS 84	DQ103887			Strain	Jiang J et al.
30.		*I. baumii*	SFC 050511–32	AY972811			Strain	Jung HS & Lee JS
31.		*I. baumii*	SFC 050527–67	AY972812			Strain	Jung HS & Lee JS
32.		*P. baumii*	SFC 960405–4	AF534068			Strain	Lim YW et al.
33.		*S. baumii*	SFCC 50029	AY558608			Strain	Jeong WJ et al.
34.		*I. baumii*	SH 3	FJ190412			Strain	Zou L et al.
35.		*S. baumii*	Yuan 4909	KY328310^a^	Angiosperm	Heilongjiang, China	Specimen	Zhu L & Cui BK
36.		*S. baumii*	Yuan 4929	KY328306^a^	*Alnus*	Heilongjiang, China	Specimen	Zhu L & Cui BK
37.	*S. ligneus*	*S. ligneus*	MG 12	KR073081^a^	*Lonicera caucasica*	Iran	Strain	Ghobad-Nejhad M
38.		*S. ligneus*	MG 13	KR073082^a^	*Lonicera caucasica*	Iran	Strain	Ghobad-Nejhad M
39.	*S. lonicericola*	*I. baumii*	BM-3753	HQ845063		China	Strain	Hu W & Deng X
40.		*I. baumii*	BM-8335	HQ845064		China	Strain	Hu W & Deng X
41.		*S. lonicericola*	Cui 10994	MF772786^a^		China	Specimen	Zhu L & Cui BK
42.		*I. lonicericola*	Dai 8322	JN642571^a^	*Lonicera*	Heilongjiang, China	Specimen	Wu SH et al.
43.		*I. lonicericola*	Dai 8335	JN642573^a^	*Lonicera*	Heilongjiang, China	Specimen	Wu SH et al.
44.		*I. lonicericola*	Dai 8340	JN642574^a^	*Lonicera*	Heilongjiang, China	Specimen	Wu SH et al.
45.		*I. lonicericola*	Dai 8376	JQ860308^a^	*Lonicera*	Heilongjiang, China	Specimen	Tian XM et al.
46.		***S. lonicericola***	**Dai 17304 (BJFC)**	**MT348582**^a^	***Lonicera***	**Liaoning, China**	**Strain**	**This study**
47.		*P.* sp.	HN100K9	KF589300		South Korea	Strain	Kang HW & Kim JK
48.		*P. ribis*	SFCC 50032	AY558643			Strain	Jeong WJ et al.
49.		*I. lonicericola*	TAA 105317	JN642572^a^	*Lonicera ruprechtiana*	Russian Far East	Specimen	Wu SH et al.
50.	*S. lonicerinus*	*S. lonicerinus*	Dai 17093	MF772788^a^	*Lonicera*	Uzbekistan	Specimen	Zhu L & Cui BK
51.		*S. lonicerinus*	Dai 17095	MF772787^a^	*Lonicera*	Uzbekistan	Specimen	Zhu L & Cui BK
52.		*S. lonicerinus*	MG 280	KU213573^a^			Specimen	Langer EJ & Ghobad-Nejhad M
53.		*S. lonicerinus*	MG 281	KU213574^a^			Specimen	Langer EJ & Ghobad-Nejhad M
54.		*I.* sp.	TAA 55428	JN642575^a^	*Lonicera*	Turkmenistan	Strain	Wu SH et al.
55.		***S. lonicerinus***	**TAA 55696**	**MT348583**^a^	***Lonicera***	**Turkmenistan**	**Specimen**	**This study**
56.		*P. linteus*	TAA-104264	AF534074			Strain	Lim YW et al.
57.	*S. microcystideus*	*S. microcystideus*	O 915609	KP030787^a^	*Olea africana*	Tanzania	Specimen	Zhou LW et al.
58.	*S. pilatii*	*P. pilatii*	BRNM 771989	KT428764^a^	*Populus alba*	Czech Republic	Specimen	Tomšovský M
59.	*S. quercicola*	*P. rhabarbarinus*	CBS 282.77	AY558642			Strain	Jeong WJ et al.
60.		*S. quercicola*	Dai 13947	KY328309^a^		Chongqing, China	Specimen	Zhu L & Cui BK
61.		*S. quercicola*	Li 445	KY328311^a^	Angiosperm	Henan, China	Specimen	Zhu L & Cui BK
62.		*S. quercicola*	Li 1149	KY328312^a^	*Quercus*	Henan, China	Specimen	Zhu L & Cui BK
63.		***S. quercicola***	**LWZ 20170821–13 (IFP)**	**MT348584**^a^	**Angiosperm**	**Hubei, China**	**Specimen**	**This study**
64.		***S. quercicola***	**LWZ 20170821–14 (IFP)**	**MT348585**^a^	**Angiosperm**	**Hubei, China**	**Specimen**	**This study**
65.		***S. quercicola***	**LWZ 20170821–18 (IFP)**	**MT348586**^a^	**Angiosperm**	**Hubei, China**	**Specimen**	**This study**
66.		***S. quercicola***	**Wei 7575 (IFP)**	**MT348587**^a^	***Quercus***	**Henan, China**	**Strain**	**This study**
67.		*S.* sp.	Wu 1805–2	MK400422^a^	*Toxicodendron*	Hubei, China	Specimen	Wu SH et al.
68.		*S.* sp.	Wu 1805–3	MK400423^a^	*Toxicodendron*	Hubei, China	Specimen	Wu SH et al.
69.		*S.* sp.	Wu 1805–5	MK400424^a^	*Toxicodendron*	Hubei, China	Specimen	Wu SH et al.
70.		*S.* sp.	Wu 1807–2	MK729538^a^	*Toxicodendron*	Hubei, China	Specimen	Wu SH et al.
71.		*S.* sp.	Wu 1807–3	MK729540^a^	*Toxicodendron*	Hubei, China	Specimen	Wu SH et al.
72.		*S.* sp.	Wu 1807–4	MK729539^a^	*Toxicodendron*	Hubei, China	Specimen	Wu SH et al.
73.	*S. sanghuang*	*I. baumii*		KM385537		Viet Nam	Strain	Hanh VV & Nguyet NT
74.		***S. sanghuang***	**AH1 (HMAS)**	**MT421899**^a^	**Cultivated**	**Anhui, China**	**Strain**	**This study**
75.		***S. sanghuang***	**AH2 (HMAS)**	**MT421900**^a^	**Cultivated**	**Anhui, China**	**Strain**	**This study**
76.		***S. sanghuang***	**AH3 (HMAS)**	**MT421901**^a^	**Cultivated**	**Anhui, China**	**Strain**	**This study**
77.		***S. sanghuang***	**AH4 (HMAS)**	**MT421902**^a^	**Cultivated**	**Anhui, China**	**Strain**	**This study**
78.		***S. sanghuang***	**AH5 (HMAS)**	**MT421903**^a^	**Cultivated**	**Anhui, China**	**Strain**	**This study**
79.		*P. igniarius*	ASI 26010	KT862134		Jeongseon, South Korea	Strain	Han JG et al.
80.		*T. linteus*	ASI 26011	KT862135		India	Strain	Han JG et al.
81.		*T. linteus*	ASI 26016	KT862136		South Korea	Strain	Han JG et al.
82.		*T. linteus*	ASI 26021	KT862138		Hongcheon, South Korea	Strain	Han JG et al.
83.		*T. linteus*	ASI 26022	KT862139		Hongcheon, South Korea	Strain	Han JG et al.
84.		*T. linteus*	ASI 26025	KT862140		Wonju, South Korea	Strain	Han JG et al.
85.		*T.linteus*	ASI 26026	KT862141		Wonju, South Korea	Strain	Han JG et al.
86.		*T. linteus*	ASI 26039	KT862143		Pyeongchang, South Korea	Strain	Han JG et al.
87.		*T. linteus*	ASI 26046	KT862144		Hongcheon, South Korea	Strain	Han JG et al.
88.		*T. linteus*	ASI 26049	KT862145		Hongcheon, South Korea	Strain	Han JG et al.
89.		*T. linteus*	ASI 26054	KT862147		Hongcheon, South Korea	Strain	Han JG et al.
90.		*T. linteus*	ASI 26062	KT862148		Hwacheon, South Korea	Strain	Han JG et al.
91.		*T. linteus*	ASI 26063	KT862149		Jeongseon, South Korea	Strain	Han JG et al.
92.		*T. linteus*	ASI 26066	KT862150		Inje, South Korea	Strain	Han JG et al.
93.		*T. linteus*	ASI 26067	KT862151		Inje, South Korea	Strain	Han JG et al.
94.		*T. linteus*	ASI 26070	KT862152			Strain	Han JG et al.
95.		*T. linteus*	ASI 26071	KT862153			Strain	Han JG et al.
96.		*T. linteus*	ASI 26073	KT862154		South Korea	Strain	Han JG et al.
97.		*T. linteus*	ASI 26074	KT862155		Seongnam, South Korea	Strain	Han JG et al.
98.		*T. linteus*	ASI 26082	KT862156		Mokpo, South Korea	Strain	Han JG et al.
99.		*T. linteus*	ASI 26088	KT862159		Sancheong, South Korea	Strain	Han JG et al.
100.		*T. linteus*	ASI 26114	KT862164		South Korea	Strain	Han JG et al.
101.		*T. linteus*	ASI 26115	KT862165		South Korea	Strain	Han JG et al.
102.		*P. linteus*	ATCC 26710	AF153010		South Korea	Strain	Kim GY et al.
103.		*S. sanghuang*	Batch 1-12192170-1	KT693244	Purchased	USA	Strain	Raja HA et al.
104.		*S. sanghuang*	Batch 2-10221252-2	KT693275	Purchased	USA	Strain	Raja HA et al.
105.		*S. sanghuang*	Batch 2-12192170-1	KT693246	Purchased	USA	Strain	Raja HA et al.
106.		***S. sanghuang***	**BJ (HMAS)**	**MT421904**^a^	**Cultivated**	**Beijing, China**	**Strain**	**This study**
107.		*I.* sp.	BZ-A	JN642589^a^	*Morus*	Hunan, China	Strain	Wu SH et al.
108.		*I.* sp.	BZ-C	JN642587^a^	*Morus*	Hunan, China	Strain	Wu SH et al.
109.		*I.* sp.	CA	JN642579^a^	*Morus*	Jiangxi, China	Strain	Wu SH et al.
110.		*I.* sp.	CB	JN642580^a^	*Morus*	Jiangxi, China	Strain	Wu SH et al.
111.		*I.* sp.	CC	JN642581^a^	*Morus*	Jiangxi, China	Strain	Wu SH et al.
112.		*S. sanghuang*	Cui 14419	MF772789^a^	*Morus*	Shaanxi, China	Specimen	Zhu L & Cui BK
113.		*S. sanghuang*	Cui 14420	MF772790^a^	*Morus*	Shaanxi, China	Specimen	Zhu L & Cui BK
114.		*I. sanghuang*	Dai 12723	JQ860316^a^	*Morus*	Sichuan, China	Specimen	Tian XM et al.
115.		***S. sanghuang***	**DB1 (HMAS)**	**MT421905**^a^	**Cultivated**	**Northeast China**	**Strain**	**This study**
116.		*P. linteus*	DGUM25003	AF082102			Strain	Chung JW et al.
117.		*P. linteus*	DGUM25004	AF080458			Strain	Chung JW et al.
118.		*I. linteus*	FS 656160	GU903004			Strain	Yu TW
119.		*I. linteus*	FS 656161	HM584806			Strain	Yu TW
120.		*T. linteus*	FS 656179	KU867779			Strain	Yu TW
121.		*T. linteus*	FS 656180	KU867780			Strain	Yu TW
122.		***S. sanghuang***	**HB (HMAS)**	**MT421907**^a^	**Cultivated**	**Hubei, China**	**Strain**	**This study**
123.		*P. linteus*	IFO 6980	AF200226			Strain	Kim GY & Lee JD
124.		*I. linteus*	IFO 6989	AY640937			Strain	Lee JS & Jung HS
125.		*P. linteus*	IMSNU 31014	AF082101			Strain	Chung JW et al.
126.		*S. sanghuang*	JL-01	MG062789			Strain	Xu X
127.		***S. sanghuang***	**JS1 (HMAS)**	**MT421908**^a^	**Cultivated**	**Jiangsu, China**	**Strain**	**This study**
128.		*I. linteus*	KAB-PL-01	DQ462333		Taiwan, China	Strain	Chiou SJ & Yen JH
129.		*P. linteus*	KCTC 6190	AF077678			Strain	Chung JW et al.
130.		*P. igniarius*	KCTC 16890	AY189708			Strain	Nam BH et al.
131.		*I. linteus*	KFDA 016	AY436626			Strain	Yun JC et al.
132.		*I. linteus*	KFDA P38	AY513234			Strain	Jin CY et al.
133.		*I. linteus*	KSSW01	EF506943			Strain	Park SY et al.
134.		*I. linteus*	LT-0802	HQ845059		South Korea	Strain	Hu W & Deng X
135.		*I. linteus*	LT-CBS83	HQ845060		South Korea	Strain	Hu W & Deng X
136.		***S. sanghuang***	**LWZ 20180927–3 (HMAS)**	**MT348588**^a^	***Morus***	**Yunnan, China**	**Specimen**	**This study**
137.		*P. linteus*	MPNU 7016	AF153009			Strain	Kim GY et al.
138.		*I. linteus*	MUCL 47139	GU461973		Cuba	Strain	Amalfi M et al.
139.		*I. linteus*	NAAS00002	JN043317			Strain	Seok SJ et al.
140.		*P. linteus*	Namsan No1	AF080457			Strain	Chung JW et al.
141.		*I. linteus*	PL 0801	FJ940906			Strain	Xie LY et al.
142.		*I. linteus*	PL 5	EF095712			Strain	Park BW et al.
143.		*I.* sp.	PL 10	JN642588^a^		China	Strain	Wu SH et al.
144.		*S. sanghuang*	S3	MN153568			Strain	Song JL et al.
145.		*P.* sp.	SA 01	EF694971			Strain	Zeng NK et al.
146.		*P. baumii*	SFC 20001106–1	AF534064			Strain	Lim YW et al.
147.		*P. baumii*	SFC 20010212–1	AF534062			Strain	Lim YW et al.
148.		*S. sanghuang*	SS	MG209821			Strain	Cai C & Zhao G
149.		*I.* sp.	T004	JN642586^a^	*Morus*	Taiwan, China	Strain	Wu SH et al.
150.		*I.* sp.	TH	JN642582^a^	*Morus*	Taiwan, China	Strain	Wu SH et al.
151.		*I.* sp.	TJ	JN642585^a^	*Morus*	Taiwan, China	Strain	Wu SH et al.
152.		*I.* sp.	TM	JN642583^a^	*Morus*	Taiwan, China	Strain	Wu SH et al.
153.		*I.* sp.	TN	JN642584^a^	*Morus*	Taiwan, China	Strain	Wu SH et al.
154.		*I.* sp.	WD 1222	JN642576^a^	*Morus*	Japan	Strain	Wu SH et al.
155.		*I.* sp.	WD 2261	JN642577^a^	*Morus*	Japan	Strain	Wu SH et al.
156.		*I.* sp.	WD 2300	JN642578^a^	*Morus*	Japan	Strain	Wu SH et al.
157.		*I.* sp.	Wu 0903–1	JN794061^a^	*Morus*	Jilin, China	Strain	Wu SH et al.
158.		*I.* sp.	ZhangjiaJie	MN242716	Cultivated		Strain	Wang Y
159.		***S. sanghuang***	**ZJ1 (HMAS)**	**MT421910**^a^	**Cultivated**	**Zhejiang, China**	**Strain**	**This study**
160.		***S. sanghuang***	**ZJ2 (HMAS)**	**MT421911**^a^	**Cultivated**	**Zhejiang, China**	**Strain**	**This study**
161.		***S. sanghuang***	**ZJ4 (HMAS)**	**MT421913**^a^	**Cultivated**	**Zhejiang, China**	**Strain**	**This study**
162.		***S. sanghuang***	**ZJ5 (HMAS)**	**MT421914**^a^	**Cultivated**	**Zhejiang, China**	**Strain**	**This study**
163.	*S. subbaumii*	*I. baumii*	BZ-2029	JN642565	Pruchased	China	Strain	Wu SH et al.
164.		*I. baumii*	BZ-2030	JN642566	Pruchased	China	Strain	Wu SH et al.
165.		***S. subbaumii***	**Dai 13360 (BJFC)**	**MT343580**^a^	***Prunus***	**Shanxi, China**	**Specimen**	**This study**
166.		***S. subbaumii***	**LWZ 20190722–18 (HMAS)**	**MT348581**^a^	**Angiosperm**	**Beijing, China**	**Specimen**	**This study**
167.		*P. linteus*	SFC 970527–1	AF534073			Strain	Lim YW et al.
168.		*I. baumii*	Wu 0910–54	JN642570^a^	*Syringa*	Beijing, China	Strain	Wu SH et al.
169.		*I. baumii*	Yuan 2444	JX069836^a^	Angiosperm	Shanxi, China	Specimen	Tian XM et al.
170.	*S. vaninii*	*I. vaninii*		HQ845058		China	Strain	Hu W & Deng X
171.		*I.* sp.	BeiJing	MN242720	Cultivated	China	Strain	Wang Y
172.		*I. vaninii*	BZ-2031	JN642593^a^	*Populus*	China	Strain	Wu SH et al.
173.		*I. vaninii*	CJC 01	JN642592^a^	Cultivated	Taiwan, China	Strain	Wu SH et al.
174.		*S. vaninii*	Cui 9939	MF772792^a^		Jilin, China	Specimen	Zhu L & Cui BK
175.		*S. vaninii*	Cui 14082	MF772793^a^	*Populus*	Jilin, China	Specimen	Zhu L & Cui BK
176.		*I. vaninii*	Dai 3624	JN642590^a^	*Populus*	China	Strain	Wu SH et al.
177.		*I. vaninii*	Dai 7011	JN642591^a^	*Populus davidiana*	Jilin, China	Strain	Wu SH et al.
178.		*S. vaninii*	Dai 8236	MF772791^a^	*Populus*	Jilin, China	Specimen	Zhu L & Cui BK
179.		***S. vaninii***	**DB2 (HMAS)**	**MT421906**^a^	**Cultivated**	**Northeast China**	**Strain**	**This study**
180.		*I. baumii*	FS 656170	GU903008			Strain	Yu TW
181.		*F. gilva*	FS 656175	HM584811			Strain	Yu TW
182.		*S. vaninii*	HZ-01	MG062791			Strain	Xu X
183.		*I.* sp.	JinZhai	MN242717	Cultivated	China	Strain	Wang Y
184.		***S. vaninii***	**JS2 (HMAS)**	**MT421909**^a^	**Cultivated**	**Jiangsu, China**	**Strain**	**This study**
185.		*I.* sp.	KangNeng	MN242721	Cultivated	China	Strain	Wang Y
186.		*I. baumii*	KFDA 015	AY436623			Strain	Yun JC et al.
187.		*I. baumii*	KFDA 022	AY436624			Strain	Yun JC et al.
188.		*I. linteus*	KFDA 024	AY436627			Strain	Yun JC et al.
189.		*I. baumii*	KFDA 029	AY436625			Strain	Yun JC et al.
190.		*I. baumii*	KFDA P36	AY509198			Strain	Jin CY et al.
191.		*I. baumii*	KFDA P40	AY509199			Strain	Jin CY et al.
192.		*I. baumii*	KFDA P45	AY509201			Strain	Jin CY et al.
193.		*I.* sp.	Korea	MN242719	Cultivated	China	Strain	Wang Y
194.		*S. baumii*	LC 6686	MK818502			Strain	Li ZN
195.		*I. linteus*	LT-HG	HQ845061			Strain	Hu W & Deng X
196.		*F. gilva*	MDJCBS87	DQ103884			Strain	Jiang J et al.
197.		*P. baumi*	MPNU 7004	AF200229			Strain	Kim GY & Lee JD
198.		*P. baumi*	MPNU 7005	AF200230			Strain	Kim GY & Lee JD
199.		*P. baumi*	MPNU 7006	AF200231			Strain	Kim GY & Lee JD
200.		*P.* sp.	MPNU 7007	AF200235			Strain	Kim GY & Lee JD
201.		*P.* sp.	MPNU 7010	AF153007		South Korea	Strain	Kim GY et al.
202.		*P.* sp.	MPNU 7012	AF153008		South Korea	Strain	Kim GY et al.
203.		*P.* sp.	MPNU 7013	AF153011		South Korea	Strain	Kim GY et al.
204.		*I. baumii*	PB 0802	FJ940907			Strain	Xie LY et al.
205.		*I. baumii*	PB 0803	FJ940908			Strain	Xie LY et al.
206.		*I. baumii*	PB 0806	FJ940911			Strain	Xie LY et al.
207.		*I. baumii*	PB 0808	FJ940913			Strain	Xie LY et al.
208.		*I. baumii*	PB 0809	FJ940914			Strain	Xie LY et al.
209.		*I.* sp.	QianDaoHu	MN242718	Cultivated	China	Strain	Wang Y
210.		*S. vaninii*	S1	MN153566			Strain	Song JL et al.
211.		*S. baumii*	S2	MN153567			Strain	Song JL et al.
212.		*F. gilva*	S12	MT275660	*Morus*	Zhejiang, China	Strain	Li Y & Huo J
213.		*P.* sp.	SA 02	EF694972			Strain	Zeng NK et al.
214.		*P.* sp.	SA 03	EF694973			Strain	Zeng NK et al.
215.		*P.* sp.	SA 04	EF694974			Strain	Zeng NK et al.
216.		*I. baumii*	SA 05	EF694975			Strain	Zeng NK et al.
217.		*P.* sp.	SA 06	EF694976			Strain	Zeng NK et al.
218.		*P.* sp.	SA 07	EF694977			Strain	Zeng NK et al.
219.		*P. linteus*	SFC 970605	AF534071			Strain	Lim YW et al.
220.		*P. linteus*	SFC 20001106–7	AF534070			Strain	Lim YW et al.
221.		*P. baumii*	SFC 20010212–2	AF534063			Strain	Lim YW et al.
222.		*T. linteus*	SFCC 10209	AY558628			Strain	Jeong WJ et al.
223.		*F. gilva*	SH 1	FJ190410			Strain	Zou L et al.
224.		*I. baumii*	SJ	JN887691			Strain	Shin KS
225.		*I. vaninii*	Wei 3382	JN169788^a^		Jilin, China	Specimen	Zhou LW & Qin WM
226.		*I. vaninii*	WN 0801	HQ845054		China	Strain	Hu W & Deng X
227.		*I. vaninii*	WN-1	HQ845055		China	Strain	Hu W & Deng X
228.		*I. vaninii*	WN-2	HQ845056		China	Strain	Hu W & Deng X
229.		*I. vaninii*	WN-4	HQ845065		China	Strain	Hu W & Deng X
230.		*I. vaninii*	WN 8213	HQ845052		China	Strain	Hu W & Deng X
231.		*I. vaninii*	WN 8824	HQ845051		China	Strain	Hu W & Deng X
232.		*I. vaninii*	WN 3624	HQ845050		China	Strain	Hu W & Deng X
233.		*S. baumii*	XZ-01	MG062790			Strain	Xu X
234.		*I. baumii*	YC	JN887692			Strain	Shin KS
235.		*S. vaninii*	Yuan 2764	KY328308^a^	*Quercus*	Shaanxi, China	Specimen	Zhu L & Cui BK
236.		*S. vaninii*	Yuan 5604	KY328307^a^	*Quercus*	Jilin, China	Specimen	Zhu L & Cui BK
237.		***S. vaninii***	**ZJ3 (HMAS)**	**MT421912**^a^	**Cultivated**	**Zhejiang, China**	**Strain**	**This study**
238.	*S. weigelae*	*S. weigelae*	420526MF0201	MH142013		Hubei, China	Specimen	Wang R et al.
239.		*I. weigelae*	Cui 6010	JQ860318^a^	*Lonicera*	Jiangxi, China	Specimen	Tian XM et al.
240.		*I. weigelae*	Cui 6012	JQ860319^a^	*Lonicera*	Jiangxi, China	Specimen	Tian XM et al.
241.		*I. weigelae*	Cui 7176	JQ860320^a^	*Syringa*	Hebei, China	Specimen	Tian XM et al.
242.		*I. weigelae*	Dai 6352	JQ860317^a^		Zhejiang, China	Specimen	Tian XM et al.
243.		*I. weigelae*	Dai 11694	JQ860315^a^		Hunan, China	Specimen	Tian XM et al.
244.		*S. weigelae*	Dai 15770	MF772795^a^	*Weigela*	Chongqing, China	Specimen	Zhu L & Cui BK
245.		***S. weigelae***	**Dai 16072 (BJFC)**	**MT348589**^a^	***Weigela***	**Inner Mongolia, China**	**Specimen**	**This study**
246.		*S. weigelae*	Dai 16077	MF772794^a^	*Weigela*	Inner Mongolia, China	Specimen	Zhu L & Cui BK
247.		***S. weigelae***	**LWZ 20150802–3 (IFP)**	**MT348590**^a^	***Weigela***	**Jiangxi, China**	**Specimen**	**This study**
248.		***S. weigelae***	**LWZ 20150802–5 (IFP)**	**MT348591**^a^	***Weigela***	**Jiangxi, China**	**Specimen**	**This study**
249.		*P. baumii*	SFC 20000111–10	AF534067			Strain	Lim YW et al.
250.		*I.* sp.	WD 1186	JN642597^a^	*Weigela*	Japan	Strain	Tian XM et al.
251.		*I.* sp.	WD 1187	JN642598^a^	*Weigela*	Japan	Strain	Tian XM et al.
252.		*I.* sp.	WD 1667	JN642594^a^	*Weigela cordeenis*	Japan	Strain	Wu SH et al.
253.		*I.* sp.	WD 1837	JN642595^a^	*Weigela cordeenis*	Japan	Strain	Wu SH et al.
254.		*I.* sp.	WD 1838	JN642596^a^	*Weigela cordeenis*	Japan	Strain	Wu SH et al.
255.		*I. weigelae*	Wei 2120	JQ860314^a^	*Coriaria*	Hubei, China	Specimen	Tian XM et al.
256.		*I. weigelae*	Wei 2267	JX069835^a^	Angiosperm	Hubei, China	Specimen	Tian XM et al.
257.		*I. tenuicontextus*	Yuan 5526	JN169786^a^	Angiosperm	Guizhou, China	Specimen	Zhou LW & Qin WM
258.	*S. weirianus*	*S. weirianus*	CBS 618.89	AY558654^a^	*Juglans major*	Arizona, USA	Strain	Jeong WJ et al.
259.		*P. weirianus*	IMSNU 32021	AF110989^a^	*Juglans major*	Arizona, USA	Strain	Chung JW et al.
260.	*S. zonatus*	*I. zonatus*	Cui 6631	JQ860305^a^	Angiosperm	Hainan, China	Specimen	Tian XM et al.
261.		*I. zonatus*	Cui 8327	JX069837^a^	Angiosperm	Yunnan, China	Specimen	Tian XM et al.
262.		*I. zonatus*	Dai 10841	JQ860306^a^	Angiosperm	Hainan, China	Specimen	Tian XM et al.
263.	*S.* sp. 1	*I.* sp.	AM-08	JF895464		Ethiopia	Specimen	Assefa A et al.
264.		*I.* sp.	AM-19	JF895465		Ethiopia	Specimen	Assefa A et al.
265.		*I. linteus*	F915611	JX985739		Ethiopia	Specimen	Assefa A et al.
266.		*I. linteus*	Teng 3279	JX985738	*Xylosoma*	China	Specimen	Assefa A et al.
267.	*S.* sp. 2	*P.* sp.	DLL 2010–102	JQ673184	*Populus tremuloides*	USA	Strain	Brazee NJ et al.
268.		*S. vaninii*	DLL 2010–102	KU139197	*Populus tremuloides*	USA	Strain	Brazee NJ
269.	*S.* sp. 3	*P. baumii*	SFC 20001106–4	AF534066		South Korea	Strain	Lim YW et al.
270.	not *Sanghuangporus*	*S. baumii*	DL 101	KP974834		China	Strain	Sun T et al.
271.	not *Sanghuangporus*	*I. vaninii*	WN-3	HQ845057		China	Strain	Hu W & Deng X

*F. = Fuscoporia*, *I.* = *Inonotus*, *P.* = *Phellinus*, *S.* = *Sanghuangporus* and *T.* = *Tropicoporus*; newly sequenced specimens and strains are in bold

^a^ sequences considered to be reliable for further analysis

### Downloading sequences from GenBank

The genus name *Sanghuangporus* and the epithets of 14 *Sanghuangporus* species were used first as queries to search GenBank. Meanwhile, the reliable sequences of 14 *Sanghuangporus* species (Zhou et al. 2020) were used as queries to perform BLAST searches in GenBank. The cut-off value of similarity for the resulting sequences was set as 95%. All the ITS sequences matching these queries that had been deposited until 30 April 2020 were retrieved from GenBank (Table 1). In addition, recently published papers related to the taxonomy of *Sanghuangporus* were checked for supplementary information on collections generating these sequences (Wu et al. 2012a, 2019b; Zhou and Qin 2012; Tian et al. 2013; Ghobad-Nejhad 2015; Tomšovský 2015; Han et al. 2016; Zhou et al. 2016; Zhu et al. 2019; Huo et al. 2020; Shao et al. 2020).

### Phylogenetic analyses

Two datasets of ITS sequences were assembled, one consisting of all sequences recovered from searches of GenBank and newly generated sequences, and the other consisting of the subset of sequences originating from material identified by taxonomists. The datasets were separately aligned using MAFFT 7.110 (Katoh and Standley 2013) under the G-INS-i option (Katoh et al. 2005). All resulting alignments are deposited in TreeBASE (http://www.treebase.org; accession number S26272). jModelTest (Guindon and Gascuel 2003; Posada 2008) was used to estimate the best-fit evolutionary model for each alignment with calculations made under the corrected Akaike information criterion. Following the estimated models, Maximum Likelihood (ML) and Bayesian Inference (BI) algorithms were used to construct midpoint-rooted trees for the alignments. The ML algorithm was performed using raxmlGUI 2.0 (Stamatakis 2014; Edler et al. 2021), and the bootstrap (BS) replicates were calculated under the auto FC option (Pattengale et al. 2010). The BI algorithm was performed using MrBayes 3.2 (Ronquist et al. 2012), which employed two independent runs each with four chains and starting from random trees. Trees were sampled every 1000th generation, of which the first 25% were removed as burn-in and the other 75% were retained for constructing a 50% majority consensus tree and calculating Bayesian posterior probabilities (BPPs). Tracer 1.5 (http://tree.bio.ed.ac.uk/software/tracer/) was used to judge the convergence of the chains.

### Evaluation of molecular species delimitation

Molecular species delimitation was estimated using multi-rate Poisson Tree Processes (mPTP) method (Kapli et al. 2017). The Newick tree file generated from the ML algorithm was directly uploaded to the web-service version (https://mptp.h-its.org/#/tree) with no outgroup taxon.

### Evaluation of genetic distances of ITS sequences

The genetic distances of an alignment of ITS sequences were estimated using MEGA X (Kumar et al. 2018; Stecher et al. 2020). For genetic distances between and within species of *Sanghuangporus*, the parameters were set as follows: a BS method of variance estimation with 1000 BS replications, a *p*-distance substitution model including transitions and transversions, uniform rates among sites, and a pairwise deletion treatment of gaps and missing data.

### Identification of diagnostic ITS sequences

Identification of diagnostic ITS sequences was according to the alignment of the ITS sequences generated using MAFFT 7.110 (Katoh and Standley 2013) under the G-INS-i option (Katoh et al. 2005); if a fragment was more than one nucleotide long and was unique for one species and not variant within this species then this fragment was identified as a potential diagnostic sequence for this species.

## RESULTS

**Fig. 1:**
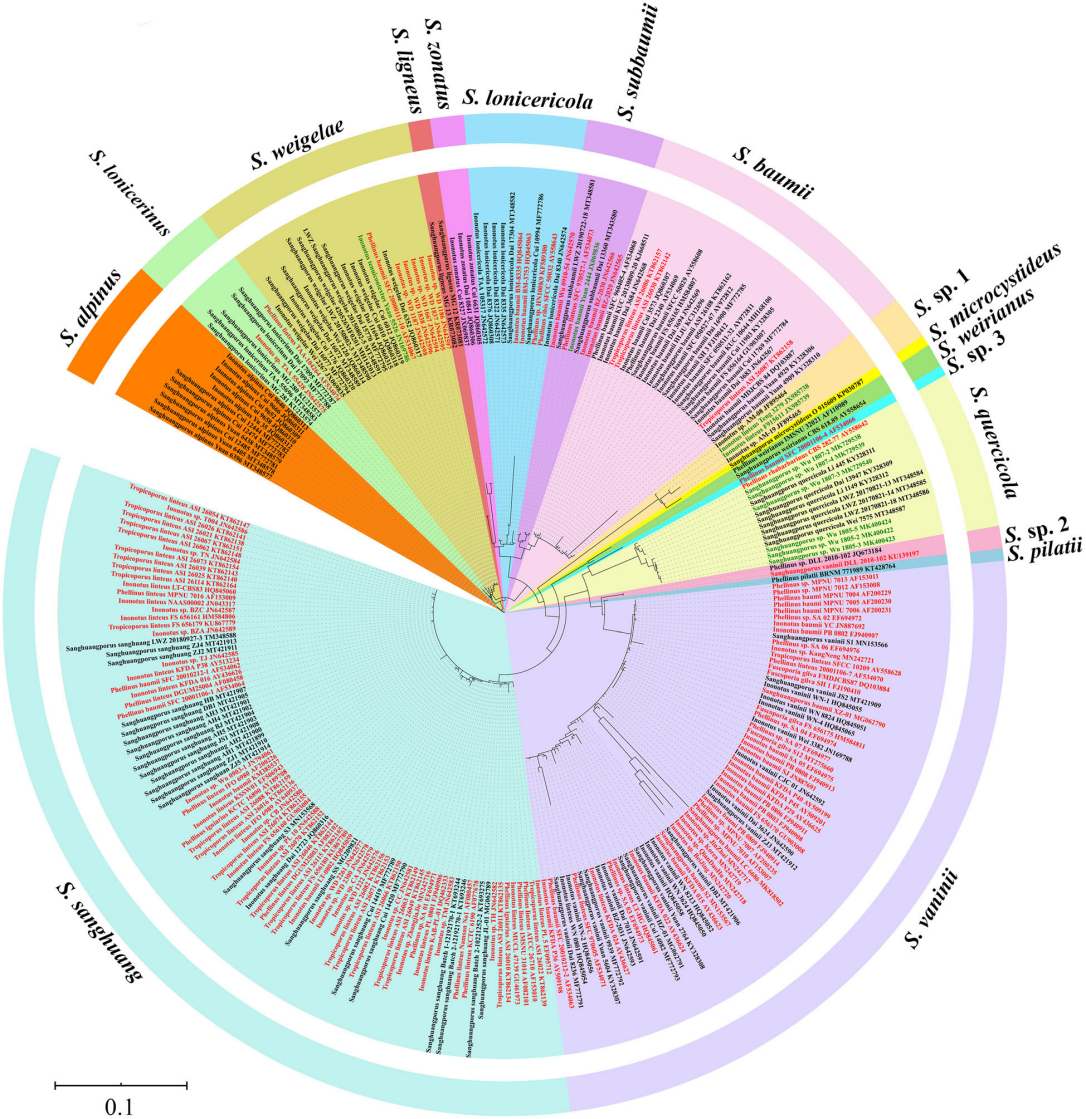
The phylogenetic tree inferred from 269 ITS sequences. The topology was generated from the maximum likelihood algorithm. The tips in green represent mislabeled specimens, while those in red represent mislabeled strains

A total of 13 specimens and 18 strains were newly sequenced, and the resulting ITS sequences were submitted to GenBank (Table 1). According to our criteria, 240 ITS sequences were downloaded from GenBank, but two sequences (HQ845057 and KP974834, originally identified as *Inonotus vaninii* and *Sanghuangporus baumii*, respectively) showed unexpectedly large differences from other sequences of *Sanghuangporus* by BLAST search, and thus were considered not to belong to the genus and were excluded from subsequent phylogenetic analyses (Table 1). Eventually, a dataset of all available 269 ITS sequences (31 newly sequenced and 238 downloaded from GenBank) from *Sanghuangporus* species was used to construct a preliminary phylogenetic framework for this genus. An alignment of 941 characters resulted from this dataset, and HKY + G was estimated as the best-fit evolutionary model for phylogenetic analysis. The ML search stopped after 850 bootstrap replicates. All chains in BI converged after ten million generations, which is indicated by the estimated sample sizes (ESSs) of all parameters above 500 and the potential scale reduction factors (PSRFs) close to 1.000. The ML and BI algorithms generated nearly congruent topologies in the main lineages (Additional file 1: Tree S1, Additional file 2: Tree S2). Therefore, only the topology from the ML algorithm is visualized in a circle form here; the midpoint-rooted tree recovered 13 species and four undescribed lineages of *Sanghuangporus* (Fig. 1). The one species gap compared with the 14 accepted species is a result of collections previously identified as *S. quercicola* and *S. toxicodendri* (this species is represented by collections Wu 1805–2, Wu 1805–3, Wu 1805–5, Wu 1807–2, Wu 1807–3 and Wu 1807–4) nesting within a single clade (Fig. 1). Of the 13 recovered species of *Sanghuangporus*, the clades of *S. lonicericola* and *S. sanghuang* did not receive good statistical support, the clade of *S. alpinus* was strongly supported just by the BI algorithm, and the other species were all strongly supported by both the ML and the BI algorithms (Additional file 1: Tree S1, Additional file 2: Tree S2). *Sanghuangporus microcystideus* merged with *S.* sp. 1 in the tree inferred from the ML algorithm (Fig. 1, Additional file 1: Tree S1), but was separated from *S.* sp. 1 in the BI tree (Additional file 2: Tree S2). The relationship between *S. microcystideus* and *S.* sp. 1 is still not clear, so we tentatively treat the specimen O 915609 as the single representative of *S. microcystideus*. One undescribed lineage including seven collections BZ-2029, BZ-2030, Dai 13360, LWZ 20190722–18, SFC 970527–1, Wu 0910–54 and Yuan 2444 showed a close relationship with *S. baumii* (Fig. 1).

**Fig. 2:**
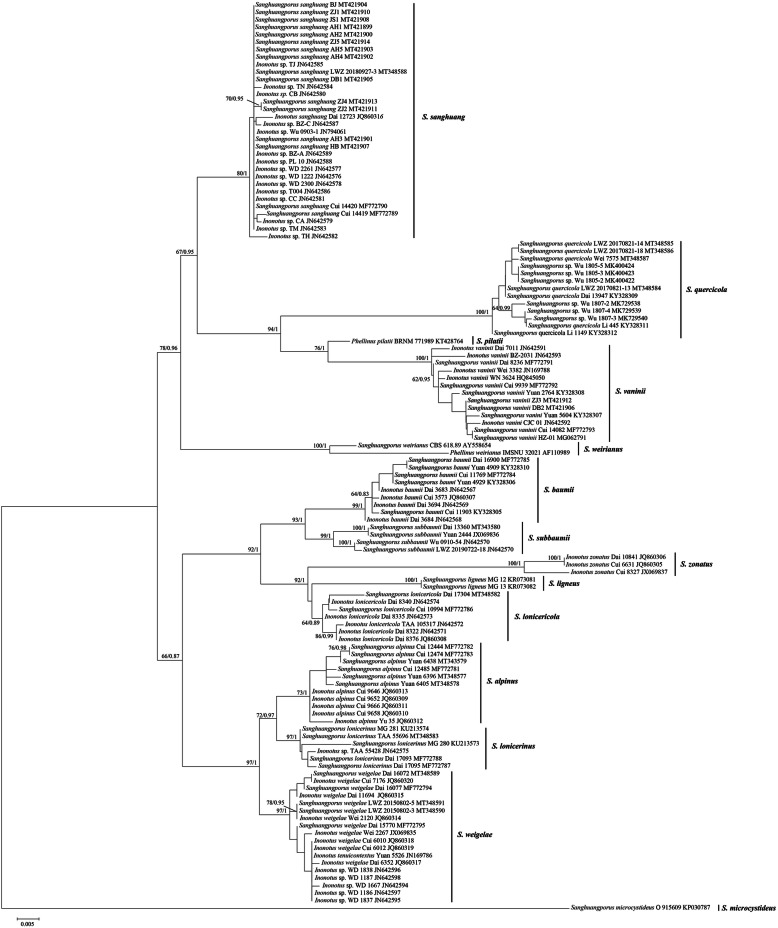
The phylogenetic tree inferred from ITS sequences submitted by taxonomists. The topology was generated from the maximum likelihood algorithm, and bootstrap values and Bayesian posterior probabilities simultaneously above 50% and 0.8, respectively, are presented at the nodes

In GenBank, species names from 10 out of 77 phylogenetically analyzed specimens were misapplied (tips labeled in green in Fig. 1), while those from 134 out of 192 phylogenetically analyzed strains were wrongly identified to species level (tips labeled in red in Fig. 1). Furthermore, two ITS sequences (HQ845057 and KP974834) of strains labeled as species of *Sanghuangporus* were extremely deviant and did not belong to the genus (Table 1). Most of these errors came from submissions by non-taxonomists. Therefore, to circumscribe species in *Sanghuangporus*, we selected the ITS sequences submitted to GenBank by taxonomists for a new round of phylogenetic analysis (Table 1). The new dataset included 122 ITS sequences and resulted in an alignment of 871 characters with HKY + I + G as the best-fit evolutionary model. The ML search stopped after 450 bootstrap replicates. All chains in BI converged after four million generations, which is indicated by the ESSs of all parameters above 1000 and the PSRFs close to 1.000. The ML and BI algorithms generated nearly congruent topologies in the main lineages, and so only the midpoint-rooted ML tree is presented along with the BPPs at the nodes (Fig. 2). As in Fig. 1, this tree also recovered 13 species of *Sanghuangporus* with *S. quercicola* and *S. toxicodendri* nested within a single clade (Fig. 2). Among these 13 species, the clade of *S. lonicericola* was still not strongly supported, and the clades of *S. alpinus* and *S. sanghuang* were moderately supported from the ML algorithm and fully supported from the BI algorithm, while the clades of all other species received strong statistical support from both the ML and the BI algorithms (Fig. 2). Moreover, in the seven collections of the undescribed lineage close to *S. baumii* in Fig. 1, four were sampled in the new dataset, and the independence of these four collections and their affinity to *S. baumii* were also strongly supported (Fig. 2). Therefore, this undescribed lineage is described as a new species, *S. subbaumii*, below.

Molecular species delimitation was estimated on the tree generated from the new dataset with 122 selected ITS sequences. The mPTP method supported the independence of 11 species, while *Sanghuangporus alpinus*, *S. lonicerinus* and *S. weigelae* were recovered as a single species (Additional file 3: Fig. S1).

To further explore the species relationships among *Sanghuangporus*, the alignment with 122 selected ITS sequences underwent a genetic distance analysis. The ranges of the within and between species genetic distances are mostly non-overlapping (Additional file 4: Table S1). *Sanghuangporus microcystideus* and *S. pilatii*, each represented by a single collection, were excluded from the within species analysis. Regarding other species of *Sanghuangporus*, the genetic distances within *S. vaninii*, *S. weirianus* and *S. zonatus* were 0–1.72%, 2.68% and 0–1.71%, respectively, whereas those within other species were no more than 1.30% and as low as 0.00% within *S. ligneus* (Additional file 4: Table S1). Regarding the genetic distances between species, all were above 1.30% except that those between *S. alpinus* and *S. lonicerinus*, and *S. baumii* and *S. subbaumii* were 1.03–2.86% and 1.19–3.07%, respectively. Across all pairwise comparisons between species, most (84 of 91) had distances above the maximum within species distance of 2.68% (Additional file 4: Table S1). Furthermore, distances between *S. microcystideus* and all other species were more than 8.90% and those between *S. pilatii* and all other species were more than 2.69% (Additional file 4: Table S1).

Based on an integrative taxonomic approach, 14 species of *Sanghuangporus* are accepted here. Their taxonomic information and reliable ITS sequences (from holotypes where possible) are provided below. Regarding *S. baumii*, *S. lonicericola*, *S. lonicerinus*, *S. microcystideus*, *S. pilatii*, *S. vaninii*, and *S. weirianus*, their holotypes were too old (50 years old or more) and so were unlikely to be successfully sequenced. Moreover, certain institutions did not make holotypes available for sequencing. Therefore, we use ITS sequences from other reference collections as reliable ITS sequences for those species.

Fifty-four ITS sequences of *S. baumii*, *S. sanghuang* and *S. vaninii*, the most common species in medicinal studies and products (Zhou et al. 2020), were further retrieved from the dataset with 122 selected sequences. These 54 sequences were realigned and the alignment is presented with shaded background (Additional file 5: Fig. S2). From this alignment, ten potential diagnostic sequences with two to six nucleotide differences were identified for HRCA to differentiate species: two for *S. baumii*, two for *S. sanghuang* and six for *S. vaninii* (Additional file 5: Fig. S2, Table 2).

**Table 2:** Diagnostic sequences with potential for discriminating *Sanghuangporus baumii*, *S. sanghuang*, and *S. vaninii* using Hyperbranched Rolling Circle Amplification. Label and position in alignment are as in Additional file 5: Fig. S2

Label	Differentiated species	Diagnostic sequence	Position in alignment	Number of diagnostic nucleotides
A	*S. sanghuang*	AWYTY	41–45	5
B	*S. vaninii*	TCA	85–87	3
C	*S. vaninii*	CTG	143–145	3
D	*S. baumii*	CGGTAGGAA	159–167	4
E	*S. vaninii*	GAGCGG	219–224	6
F	*S. vaninii*	CCCCC	264–278	4
G	*S. vaninii*	AG	556–557	2
H	*S. baumii*	AGG	650–652	2
I	*S. vaninii*	ACG	664–666	2
J	*S. sanghuang*	TT	690–691	2

## TAXONOMY

***Sanghuangporus alpinus*** (Y.C. Dai & X.M. Tian) L.W. Zhou & Y.C. Dai, *Fungal Diversity* **77**: 340 (2016).

*Basionym: Inonotus alpinus* Y.C. Dai & X.M. Tian, *Fungal Diversity* **58**: 162 (2013).

*Type*: **China:** *Tibet*: Linzhi County, Lulang, on living angiosperm tree, 24 Sept. 2010, *B.K. Cui*, *Cui 9658* (BJFC – holotype).

*ITS barcoding sequence:* JQ860310 (from holotype).

***Sanghuangporus baumii*** (Pilát) L.W. Zhou & Y.C. Dai, *Fungal Diversity* **77**: 340 (2016).

*Basionym: Phellinus baumii* Pilát, *Bull. trimest. Soc. mycol. Fr.* **48**: 25 (1932).

*Synonym: Inonotus baumii* (Pilát) T. Wagner & M. Fisch., *Mycologia* **94**: 1009 (2002).

*Type*: **Russia:** *Primorsky Krai*: Vladivostok, on trunk of *Syringae*, 5 June 1928, *M.K. Ziling 267* (PRM 189012 – holotype).

*Reference collection*: **China:** *Heilongjiang*: Yichun, Fenglin nature reserve, on living trunk of *Syringa*, 8 Sept. 2002, *Y.C. Dai*, *Dai 3683* (IFP)

*ITS barcoding sequence:* JN642567 (from the reference collection cited above, proposed by Zhou et al. (2020) and accepted here).

***Sanghuangporus ligneus*** Ghob.-Nejh., *Mycol. Progr.* **14**(90): 2 (2015).

*Type*: **Iran**: *East Azerbaijan*: Khoda-Afarin, Kalaleh-Eslami, Darana, deciduous forest with *Quercus macranthera*, *Lonicera*, *Cornus mas*, and *Crataegus*, on stem of living *Lonicera caucasica*, 10 May 2008, *M. Ghobad-Nejhad*, *Ghobad-Nejhad 1152* (ICH – holotype).

*ITS barcoding sequence:* KR073081 (from holotype).

***Sanghuangporus lonicericola*** (Parmasto) L.W. Zhou & Y.C. Dai, *Fungal Diversity* **77**: 340 (2016).

*Basionym: Phellinus lonicericola* Parmasto, *Folia cryptog. Estonica* **38**: 59 (2001).

*Synonym: Inonotus lonicericola* (Parmasto) Y.C. Dai, *Fungal Diversity* **45**: 276 (2010).

*Type*: **Russia:** *Primorsky Krai*: Lazovsky Nature Reserve, Petrov island, on trunk of *Lonicera ruprechtiana* in *Taxus* mixed forest, 2 Sept. 1961, *E. Parmasto* (TAA-M 013933 – holotype).

*Reference collection*: **China:** *Heilongjiang*: Ningan County, Jingpohu National Scenic Area, on living trunk of *Lonicera*, 8 Sept. 2007, *Y.C. Dai*, *Dai 8376* (IFP)

*ITS barcoding sequence:* JQ860308 (from the reference collection cited above, proposed by Zhou et al. (2020) and accepted here).

***Sanghuangporus lonicerinus*** (Bondartsev) Sheng H. Wu et al., *Fungal Diversity* **77**: 340 (2016).

*Basionym: Fomes lonicerinus* Bondartsev, *Acta Inst. Bot. Acad. Sci. USSR Plant. Crypt.,* Ser. II: no. 500 (1935).

*Synonyms: Phellinus lonicerinus* (Bondartsev) Bondartsev & Singer, *Annls mycol.* **39**: 56 (1941).

*Cryptoderma lonicerinum* (Bondartsev) Imazeki, *Bull. Tokyo Sci. Mus.* **6**: 107 (1943).

*Porodaedalea lonicerina* (Bondartsev) Imazeki, *Col. Ill. Mushrooms Japan*, **2**: 191 (1989).

*Inonotus lonicerinus* (Bondartsev) Sheng H. Wu et al., *Bot. Studies (Taipei)* **53**: 140 (2012).

*Type*: ***Uzbekistan*:** *Samarkand*: Sarymat, on trunk of *Lonicera tatarica*, 1926, *E. Czerniakowsk* (LE 22512 – lectotype designated by Bondartsev 1953).

*Reference collection*: **Turkmenistan:** *Bakharden*: Bakharden, Arvaz, Montes Kopet-dagh, on *Lonicera*, 17 Oct. 1971, *E. Parmasto* (TAA 55428)

*ITS barcoding sequence:* JN642575 (from the reference collection cited above, proposed by Zhou et al. (2020) and accepted here).

***Sanghuangporus microcystideus*** (Har. & Pat.) L.W. Zhou & Y.C. Dai, *Fungal Diversity* **77**: 340 (2016).

*Basionym: Phellinus microcystideus* Har. & Pat., *Bull. Mus. natn. Hist. nat.*, *Paris* **15**: 90 (1909).

*Synonym: Fomes microcystideus* (Har. & Pat.) Sacc. & Trotter, *Syll. Fung.* **21**: 286 (1912).

*Type:* **Congo:** *Moyen Oubangui*: Grande Forêt, *M.A. Chevalier 11431* (FH – holotype).

*Reference collection*: **Tanzania:** *Arusha*: Arusha National Park, Mount Meru, on trunk of *Olea africana*, 18 Feb. 1976, *R. Harjula* (O 915609)

*ITS barcoding sequence:* KP030787 (from the reference collection cited above, proposed by Zhou et al. (2020) and accepted here).

***Sanghuangporus pilatii*** (Černý) Tomšovský, *Phytotaxa* **239**: 84 (2015).

*Basionym: Phellinus pilatii* Černý, *Česká Mykol.* **22**(1): 2 (1968).

*Synonym: Porodaedalea pilatii* (Černý) Fiasson & Niemelä, *Karstenia* **24**(1): 26 (1984).

*Type:* **Czech Republic:** *Břeclav*: Tvrdonice, 8 Oct. 1955, *A. Černý* (PRM 628393 – holotype).

*Reference collection*: **Czech Republic:** *Břeclav*: Nové Mlýny, Křivé jezero National Nature Reserve, on *Populus alba*, 22 Oct. 2011, *M. Tomšovský 41/2011* (BRNM 771989)

*ITS barcoding sequence:* KT428764 (from the reference collection cited above, proposed by Zhou et al. (2020) and accepted here).

***Sanghuangporus quercicola*** Lin Zhu & B.K. Cui, *Phytotaxa* **311**: 271 (2017).

*Synonym: Sanghuangporus toxicodendri* Sheng H. Wu et al., *MycoKeys* **57**: 106 (2019).

*Type:* **China:** *Henan*: Neixiang County, Baotianman Nature Reserve, on dead tree of *Quercus*, 25 Aug. 2006, *J. Li*, *Li 1149* (BJFC – holotype).

*ITS barcoding sequence:* KY328312 (from holotype).

***Sanghuangporus sanghuang*** (Sheng H. Wu et al.) Sheng H. Wu et al., *Fungal Diversity* **77**: 340 (2016).

*Basionym: Inonotus sanghuang* Sheng H. Wu et al., *Bot. Studies (Taipei)* **53**: 140 (2012).

*Type:* **China:** *Jilin*: Baishan City, on *Morus* sp., Mar. 2009, *S.H. Wu*, *Wu 0903–1* (TNM – holotype).

*ITS barcoding sequence:* JN794061 (from holotype).

**Fig. 3:**
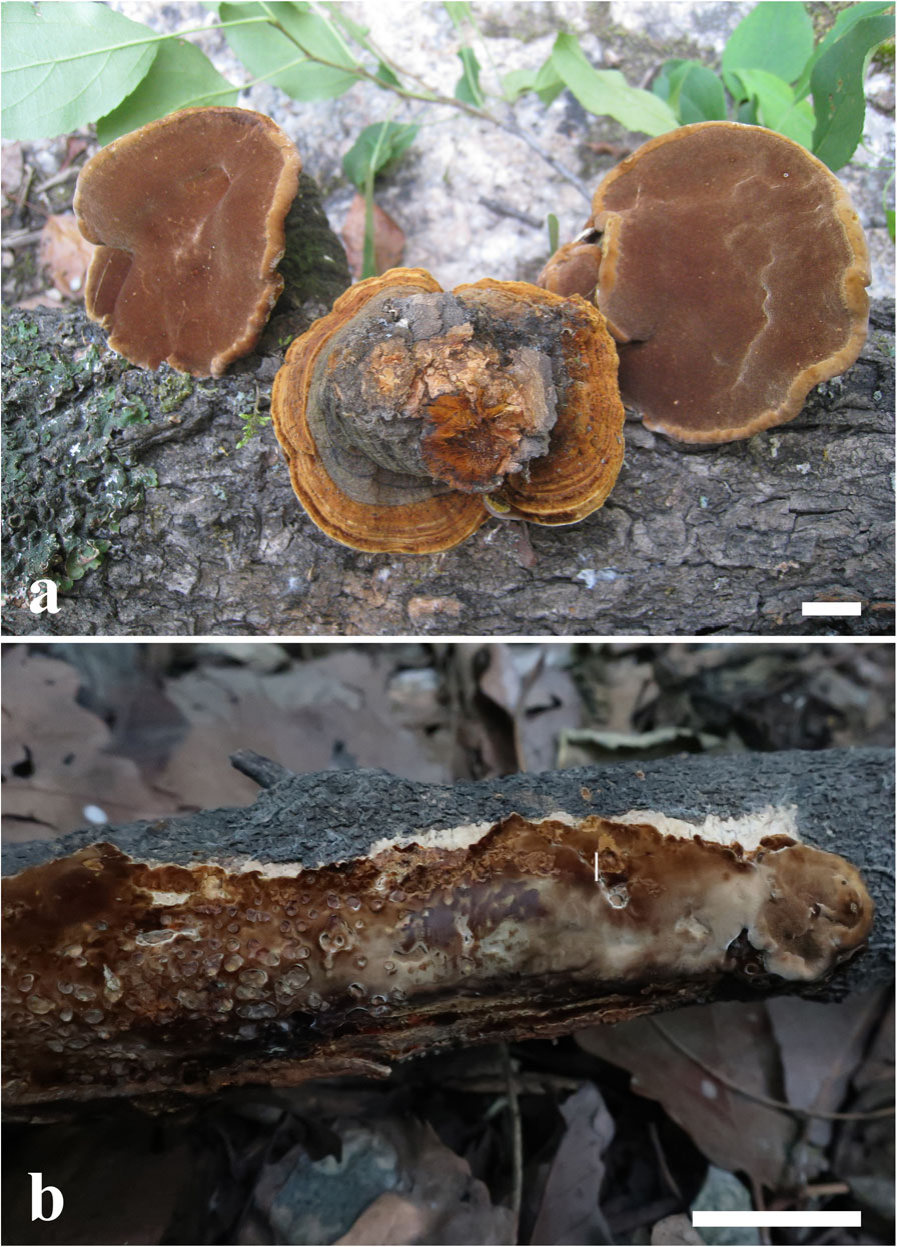
Basidiomes of *Sanghuangporus subbaumii* in situ. **a** Dai 13360 (holotype). **b** LWZ 20190722–18 (paratype). Bars: 2 cm

**Fig. 4:**
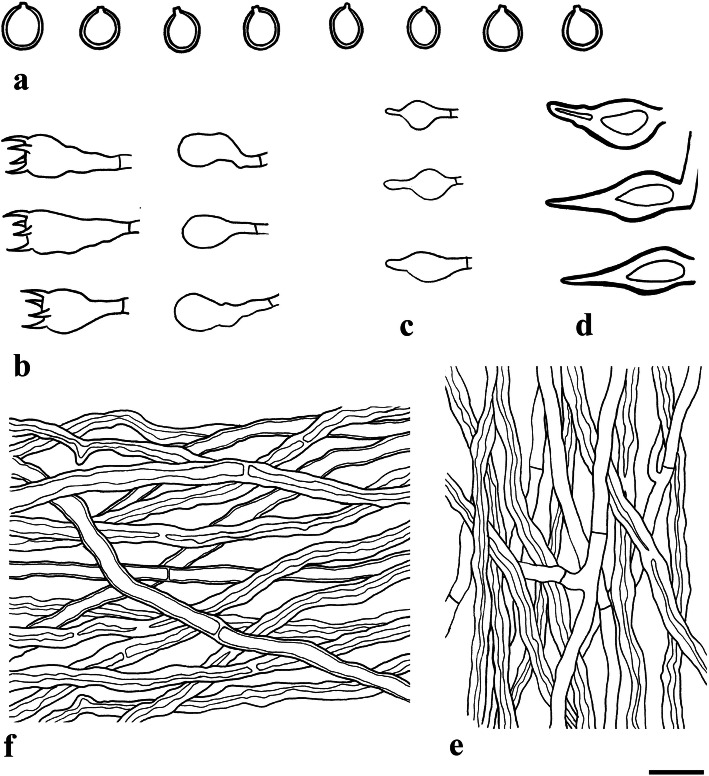
Microscopic structures of *Sanghuangporus subbaumii* (drawn from Dai 13360, holotype). **a** Basidiospores. **b** basidia and basidioles. **c** cystidioles. **d** hymenial setae. **e** hyphae from trama. **f** hyphae from context. Bars: a = 5 μm, b–e = 10 μm

***Sanghuangporus subbaumii*** Shan Shen, Y.C. Dai & L.W. Zhou, **sp. nov.** (Figs. 3 and 4).

MycoBank MB838235.

*Etymology: subbaumii* (Lat.), refers to the similarity to *Sanghuangporus baumii*.

*Diagnosis:* Differing from *S. baumii* in having resupinate, effused-reflexed to pileate basidiomes, acute pileal margin and longer hymenial setae (> 20 μm in length).

*Type:* **China:** *Shanxi*: Jiaocheng County, Pangquangou Nature Reserve, on fallen trunk of *Prunus* sp., 10 Aug. 2013, *Y.C. Dai*, *Dai 13360* (BJFC – holotype; HMAS 281653 – isotype).

*Description: Basidiomes* perennial, resupinate, effused-reflexed to pileate, without odor or taste and hard corky when fresh, woody hard when dry; to 20 cm long and 5 cm wide when resupinate. *Pilei* dimidiate, ungulate in section, projecting to 3.5 cm wide, 6 cm long and 4 cm thick at base. *Pileal surface* dark brown and velutinate when juvenile, mouse-grey to black, glabrous and cracked with age, concentrically zonate and narrowly sulcate; *margin* yellow brown, acute. *Pore surface* yellowish brown, glancing; *sterile margin* distinct, yellowish; *pores* angular to circular, 5–7 per mm; *dissepiments* thin, entire. *Context* yellowish brown to dark brown, woody hard, to 3.5 cm thick. *Tubes* yellowish brown, darker than pore surface, woody hard, to 0.5 cm long.

*Hyphal system* monomitic in context, dimitic in trama; *generative hyphae* simple septate; *tissue* darkening but otherwise unchanged in KOH. *Context* generative hyphae occasionally slightly thick-walled with a wide lumen and yellowish, mostly thick-walled with a narrow lumen and yellowish brown, unbranched, frequently septate, more or less regularly arranged, 3.5–4 μm diam. *Tubes* generative hyphae thin to slightly thick-walled, hyaline, occasionally branched, frequently septate, 3–4.5 μm diam; skeletal hyphae dominant, thick-walled with a narrow lumen, yellowish brown, unbranched, rarely septate, subparallel along the tubes, 2.2–3.7 μm diam. *Hymenial setae* frequent in the mature hymenium, subulate to ventricose, dark brown, thick-walled, 20–35 × 7–12 μm. *Cystidioles* subulate, with narrow and tapering apex, hyaline, 15–20 × 4–6 μm. *Basidia* barrel-shaped to broadly clavate, with four sterigmata and a simple septum at the base, hyaline, 20–25 × 7–9 μm; *basidioles* in shape similar to basidia, but slightly smaller. *Basidiospores* broadly ellipsoid to subglobose, yellowish, slightly thick-walled, smooth, non-amyloid, non-dextrinoid, moderately cyanophilous, (3.8–)4–4.9(− 5.2) × 3.1–3.8(− 3.9) μm, L = 4.35 μm, W = 3.41 μm, Q = 1.24–1.31 (*n* = 60/2).

*Notes: Sanghuangporus subbaumii* mostly resembles *S. baumii*, but the latter species differs in having pileate basidiomes always, obtuse pileal margin and shorter hymenial setae (< 20 μm in length; Dai 2010). The resupinate to pileate basidiomes make *S. subbaumii* similar to *S. vaninii*, but *S. vaninii* lacks cystidioles and has a thin black zone separating heterogeneous context (Dai 2010).

*ITS barcoding sequence:* MT348580 (from holotype).

*Additional specimen examined:* **China:** *Beijing*: Shangfangshan Forest Park, on fallen angiosperm trunk, 22 July 2019, *L.W. Zhou*, *LWZ 20190722–18* (HMAS 281654).

***Sanghuangporus vaninii*** (Ljub.) L.W. Zhou & Y.C. Dai, *Fungal Diversity* **77**: 340 (2016).

*Basionym: Phellinus vaninii* Ljub., *Bot. Mater.* **15**: 115 (1962).

*Synonym: Inonotus vaninii* (Ljub.) T. Wagner & M. Fisch., *Mycologia* **94**: 1009 (2002).

*Type:* **Russia:** *Primorsky Krai*: Shkotovsky District, watershed of the Maykhe river, Maykhinsky forestry, Verkhne-Maykhinskaya forest area, Peyshula, quarter 119, in valley of pine-broadleaved forest, on dried aspen tree, 14 Aug. 1951, *L.V. Lyubarskiy* (LE 22523 – holotype).

*Reference collection*: **China:** *Jilin*: Antu County, Changbaishan, on fallen trunk of *Populus davidiana*, 26 Aug. 2005, *Y.C. Dai*, *Dai 7011* (IFP)

*ITS barcoding sequence:* JN642591 (from the reference collection cited above, proposed by Zhou et al. (2020) and accepted here).

***Sanghuangporus weigelae*** (T. Hatt. & Sheng H. Wu) Sheng H. Wu et al., *Fungal Diversity* **77**: 340 (2016).

*Basionym: Inonotus weigelae* T. Hatt. & Sheng H. Wu, *Bot. Studies (Taipei)* **53**: 143 (2012).

*Synonym: Inonotus tenuicontextus* L.W. Zhou & W.M. Qin, *Mycol. Progr.* **11**: 793 (2012).

*Type:* **Japan:** *Nagano*: Chino, Minoto, *on Weigela coraeensis*, 19 Sept. 1993, *T. Hattori*, *F16899* (TFM – holotype).

*ITS barcoding sequence:* JN642596 (from holotype).

***Sanghuangporus weirianus*** (Bres.) L.W. Zhou & Y.C. Dai, *Fungal Diversity* **77**: 340 (2016).

*Basionym: Fomes weirianus* Bres., *Stud. Trent.*, Classe II, Sci. Nat. Econ. **7**(1): 5 (1926).

*Synonyms: Phellinus weirianus* (Bres.) Gilb., *J. Ariz. Acad. Sci.* **7**: 137 (1972).

*Inonotus weirianus* (Bres.) T. Wagner & M. Fisch., *Mycologia* **94**: 1009 (2002).

*Type:* **USA:** *New Mexico*: on trunk of *Juglans rupestris*, 25 Oct. 1911, *G.G. Hedgcock* & *W.H. Long* (BPI 235278 – holotype).

*Reference collection*: **USA:** *Arizona*: on *Juglans major*, 27 Aug. 1967, *R.L. Gilbertson 6975-S* (IMSNU 32021)

*ITS barcoding sequence:* AF110989 (from the reference collection cited above, proposed by Zhou et al. (2020) and accepted here).

***Sanghuangporus zonatus*** (Y.C. Dai & X.M. Tian) L.W. Zhou & Y.C. Dai, *Fungal Diversity* **77**: 341 (2016).

*Basionym: Inonotus zonatus* Y.C. Dai & X.M. Tian, *Fungal Diversity* **58**: 165 (2013).

*Type:* **China:** *Hainan*: Jianfengling Nature Reserve, on living angiosperm tree, 11 May 2009, *B.K. Cui*, *Cui 6631* (BJFC – holotype).

*ITS barcoding sequence:* JQ860305 (from holotype).

## DISCUSSION

In this study, we summarized all available ITS barcoding sequences bearing the name “Sanghuang” in GenBank. A total of 271 ITS sequences related to “Sanghuang”, including 31 newly generated sequences from this study, were analyzed. In association with previous information of morphology, hosts, and multilocus-based phylogeny, 14 species are accepted as members of *Sanghuangporus* including the new species *S. subbaumii* described herein. We also synonymize *S. toxicodendri* under *S. quercicola*.

*Sanghuangporus subbaumii* has a phylogenetically close relationship to *S. baumii*; however, these two species form two distinct lineages with strong support (Additional file 1: Tree S1, Additional file 2: Tree S2, Fig. 2). Moreover, *S. subbaumii* and *S. baumii* were also estimated as two independent species using the mPTP method (Additional file 3: Fig. S1), and for ITS the interspecific distance is 1.19–3.07%, generally above the cut-off value of interspecific distances (1.30%) within *Sanghuangporus* (Additional file 4: Table S1). Besides molecular evidence, morphological differences between these two species are also clear. Geographically, *S. subbaumii* is only known from North China, whereas Chinese collections of *S. baumii* are distributed in north-east China (Table 1).

*Sanghuangporus toxicodendri* was recently described from specimens collected from *Toxicodendron* sp. in Hubei, central China (Wu et al. 2019b) and resembles *S. quercicola*, another species originally described from central China (Zhu et al. 2017). However, in the publication introducing *S. toxicodendri* (Wu et al. 2019b) the separation from *S. quercicola* was not well-supported phylogenetically. Moreover, the morphological differences between these two species are slight (such as for basidiospore length) or involve variable characters that do not have taxonomic signal (such as the surface color of the pileal margin) (Zhu et al. 2017; Wu et al. 2019b). In the current phylogenetic analyses, the six specimens of *S. toxicodendri*, three specimens of *S. quercicola* and four additional collections merged in a fully supported clade (Additional file 1: Tree S1, Additional file 2: Tree S2, Fig. 2). The mPTP-based estimation of species delimitation also treated *S. toxicodendri* and *S. quercicola* as a single species (Additional file 3: Fig. S1) and the intraspecific distances among ITS sequences under both names were 0–1.11%, well below the threshold of 1.30% (Additional file 4: Table S1). Therefore, *S. toxicodendri* and *S. quercicola* are considered conspecific, and *S. quercicola* has priority by publication date over *S. toxicodendri*.

The clade of *S. lonicericola* was present but not well-supported in our phylogenetic analyses (Additional file 1: Tree S1, Additional file 2: Tree S2, Fig. 2). Similarly, the clades of *S. alpinus* and *S. sanghuang* were not strongly supported by the ML algorithm **(**Fig. 2). For *S. lonicericola* and *S. alpinus*, despite the lack of support in one or both analyses, each formed a distinct clade, and for both species distances to other species were above the threshold of 1.30% (*S. lonicericola* minimum 2.19% and *S. sanghuang* minimum 2.90%; Additional file 4: Table S1). In addition, *S. alpinus*, *S. lonicerinus*, and *S. weigelae*, even though forming three independent lineages, were considered conspecific by the mPTP method (Additional file 3: Fig. S1). However, the interspecific distances for ITS between *S. weigelae* and each of *S. alpinus* and *S. lonicerinus* are above the cut-off value of interspecific distances (1.30%) within *Sanghuangporus* (Additional file 4: Table S1). Regarding the pair of *S. alpinus* and *S. lonicerinus*, for ITS the between species distance (1.03–2.86%) was generally above the intraspecific distances within either species (0–1.08% and 0–1.18%, respectively; Additional file 4: Table S1). Moreover, the monophyly of *S. alpinus* was strongly supported by the BI algorithm and that of *S. lonicerinus* was strongly supported by both the ML and the BI algorithms (Fig. 2). Besides, morphological delimitations among these five species are stable (Wu et al. 2012a; Tian et al. 2013; Zhou et al. 2016). Taking all this into account, we accept *S. alpinus*, *S. lonicericola*, *S. lonicerinus*, *S. sanghuang*, and *S. weigelae* as five independent species.

*Sanghuangporus vaninii*, *S. weirianus*, and *S. zonatus* are the only three species with intraspecific ITS distances of more than 1.30% (0–1.72%, 2.68% and 0–1.71%, respectively; Additional file 4: Table S1). However, they all received strong support as independent species (Additional file 1: Tree S1, Additional file 2: Tree S2, Fig. 2, Additional file 3: Fig. S1). As one of the most commonly cultivated species, several cultivars of *S. vaninii* were included in the evaluation of genetic distances of ITS sequences (Zhou et al. 2020; Table 1). The procedure of cultivation with continuous passage culture can dramatically accelerate the accumulation of genetic variation, which may result in the higher intraspecific ITS difference in *S. vaninii*. Noteworthily, branch lengths of the only two available collections of *S. weirianus* were markedly different even though the two strains were from the same original isolate (Fig. 2). Regarding *S. zonatus*, two collections from Hainan, South China grouped together with full statistical support, and then formed a fully supported clade with a collection from Yunnan, Southwest China (Table 1, Figs. 1 and 2). Both *S. weirianus* and *S. zonatus* are poorly collected species, and a more comprehensive sampling of these two species in phylogenetic analyses will further clarify their intraspecific relationships. For now, we tentatively accept them as monophyletic species.

A study by Nilsson et al. (2006) revealed that about 10–21% of 51,000 fungal ITS sequences available at that time in the International Nucleotide Sequence Databases were annotated with incorrect taxonomic information. More recently, this proportion has increased to almost 30% (Hofstetter et al. 2019). Regarding “Sanghuang”, more than half (or say 146) of the ITS sequences labeled as such, were found to be mislabeled, implying that the proportion of incorrectly labeled ITS sequences for “Sanghuang” is much higher than the average proportion for all fungal groups. This phenomenon may be attributable to the medicinal properties of “Sanghuang”, which attracts much more attention from non-taxonomists who submit ITS sequences to GenBank. Consequently, the numerous errors result in chaos with BLAST searches, especially for non-taxonomists. Although the RefSeq Targeted Loci (RTL) database has been initiated for fungal ITS sequences from type collections (Schoch et al. 2014), only two species of *Sanghuangporus*, viz. *S. alpinus* and *S. zonatus* were reannotated and deposited under accession numbers of NR_158887 and NR_166366. Actually, ITS sequences from six holotypes of accepted *Sanghuangporus* species are available in GenBank. This number increases to eight, if two synonyms of other species of *Sanghuangporus*, viz. *Inonotus tenuicontextus* and *S. toxicodendri* are considered. In UNITE (Nilsson et al. 2019), tens of species hypotheses belonging to *Sanghuangporus* are available under various threshold values at species level; however, not all accepted species of *Sanghuangporus* (such as *S. ligneus*, *S. pilatii*, and *S. quercicola*) are referred to and the reference sequences for some species hypotheses are not always those from holotypes. Moreover, both RTL and UNITE are not familiar to mycologists working on medicinal studies and government officers in charge of the policy of medicinal fungi, who normally take the first hit of a BLAST search in GenBank as the species name. Therefore, the accuracy of ITS sequences of “Sanghuang” in GenBank is crucial for medicinal studies and commercial development of this fungal genus.

Compared with specimens, many more mislabeled ITS sequences of *Sanghuangporus* came from cultured strains, and most of those sequences were submitted by non-taxonomists. A typical case is the recent paper on genome sequencing of “Sanghuang” that also submitted six ITS sequences to GenBank (Shao et al. 2020). In GenBank, all these six sequences were labeled as *Inonotus* sp. rather than species of *Sanghuangporus* (MN242716–MN242721), while the six strains generating these sequences were named as *S. sanghuang* (Shao et al. 2020). However, five of the six strains, including the one (labeled as KangNeng) subjected to genome sequencing, are actually *S. vaninii* (Fig. 1, Zhou et al. 2020); i.e. five out of six strains were wrongly identified to species level. Therefore, this species misidentification means that the whole genome sequence of “Sanghuang” may be misapplied in future studies. Shao et al. (2020) also stated that these six strains are commercially cultivated, which further results in the name chaos for commercial products of “Sanghuang”. Another publication on genome sequencing identified the genome sequenced strain S12 as *Phellinus gilvus* according to ITS barcoding region (Huo et al. 2020). However, the corresponding ITS sequence (MT275660) annotated as *Fuscoporia gilva* in GenBank represents *S. vaninii* (Fig. 1, Zhou et al. 2020). Another case is a paper devoted to the species identity of “Sanghuang” strains (Han et al. 2016). Thirty strains deposited in the Agricultural Sciences Institute culture collection (Mushroom Research Division, Rural Development Administration, Republic of Korea) were correctly identified as *S. vaninii* and *S. sanghuang* according to an ITS-based phylogenetic analysis; however, unfortunately, most of these ITS sequences were mislabeled when being submitted to GenBank.

Ten mislabeled ITS sequences found in the current study came from basidiomes. These errors were caused mainly by taxonomic revisions of certain species. Six sequences of specimens Wu 1805–2, Wu 1805–3, Wu 1805–5, Wu 1807–2, Wu 1807–3 and Wu 1807–4 that were originally labeled as *Sanghuangporus* sp. but later cited under *S. toxicodendri* by Wu et al. (2019b) are accepted to represent *S. quercicola*. Yuan 2444, previously considered as *S. baumii*, was nested within the lineage segregated from *S. baumii* as a new species *S. subbaumii* (Figs. 1 and 2, Additional file 3: Fig. S1). Consequently, the ITS sequence of Yuan 2444 (JX069836) is corrected to *S. subbaumii* (Table 1). Another mislabeled sequence was generated from a specimen originally described as *Inonotus tenuicontextus* (Zhou and Qin 2012). Although this species was published online earlier than *Inonotus weigelae* (basionym of *S. weigelae*; Wu et al. 2012a; Tian et al. 2013), its online date is before 1 January 2012 and thus the name was not effectively published online according to Art. 29.1 of the ICNafp (Turland et al. 2018). *Inonotus tenuicontextus* was then treated as a later synonym of *I. weigelae* (Tian et al. 2013). Therefore, this mislabeled sequence is accepted to represent *S. weigelae* (Table 1).

Although intact mature basidiomes of “Sanghuang” are not difficult to identify to species level morphologically and in a short time by taxonomists working on this group, most of the commercial products are small pieces or even powders. Normally, it is impossible to rapidly determine which species those commercial products represent. As for other traditional medicinal mushrooms (Raja et al. 2017), species names of *Sanghuangporus* are sometimes misapplied to certain products of “Sanghuang” (Shao et al. 2020). This confused situation to some extent restricts the commercial development of “Sanghuang” (Zhou 2020). Therefore, to standardize the “Sanghuang” industry, ten reference sequences are provided for HRCA based on the accurate boundaries among three commonly studied and cultivated species, viz. *S. baumii*, *S. sanghuang*, and *S. vaninii* (Lin et al. 2017; Zhou et al. 2020). HRCA is an isothermal amplification approach and thus provides a rapid, simple and low-cost detection of specific nucleic acid sequences (Nilsson et al. 1994; Lizardi et al. 1998) even for single nucleotide differences (Nilsson et al. 1997). This approach has been widely used for the clinical detection of human pathogenic microfungi (Zhou et al. 2008; Trilles et al. 2014; Rodrigues et al. 2015) and, recently, was also reported for the rapid detection of poisonous macrofungi (He et al. 2019a, 2019b). Regarding lethal *Amanita* species, nucleotide differences greater than two allowed species identification using the *α-amanitin* gene (He et al. 2019a). Here, for *Sanghuangporus* a set of candidates for future testing is provided that have diagnostic sequences containing between two and six nucleotide differences.

## CONCLUSION

In order to promote medicinal studies and industrial development, the ITS barcoding region of *Sanghuangporus* species is here comprehensively analyzed to enable accurate species identification. Firstly, the ITS region is confirmed as an effective barcode in *Sanghuangporus*. Secondly, the names of all available ITS sequences in GenBank related to “Sanghuang” are carefully revised and where necessary corrected. Thirdly, the intraspecific ITS difference for each species of *Sanghuangporus* is evaluated to be up to 1.30% (except *S. vaninii*, *S. weirianus*, and *S. zonatus*), while the interspecific ITS difference is above 1.30% (except between *S. alpinus* and *S. lonicerinus*, and *S. baumii* and *S. subbaumii*). This provides a practical cut-off value for BLAST search-based species identification. Finally, ten potential diagnostic sequences are provided for HRCA assay to rapidly differentiate the three commonly studied and cultivated species, viz. *S. baumii*, *S. sanghuang*, and *S. vaninii*. As a follow up, we will suggest reannotation of ITS sequences related to “Sanghuang” to the GenBank administrators, especially to ensure that sequences from holotypes and reference collections for each species of *Sanghuangporus* are designated as such. Further, we will liaise with UNITE to ensure that appropriate reference sequences are designated for UNITE species hypotheses within *Sanghuangporus*.

## Supplementary Information

**Additional file 1: Tree S1.** The phylogenetic tree inferred from 269 ITS sequences. The topology was generated from the maximum likelihood algorithm and bootstrap values are presented at the nodes.

**Additional file 2: Tree S2.** The phylogenetic tree inferred from 269 ITS sequences. The topology was generated from the Bayesian inference algorithm and Bayesian posterior probabilities are presented at the nodes.

**Additional file 3: Figure S1.** Molecular species delimitation estimated from the Newick tree file of Fig. 2 using multi-rate Poisson Tree Processes method. The continuous red branches represent a single species.

**Additional file 4: Table S1.** Genetic distances of ITS sequences between and within species of *Sanghuangporus.*

**Additional file 5: Figure S2.** The alignment of *Sanghuangporus baumii*, *S. sanghuang* and *S. vaninii* generated from ITS sequences submitted by taxonomists. Ten potential diagnostic sequences for Hyperbranched Rolling Circle Amplification are labeled in capital letters.

## Data Availability

The materials are available as Additional files 1, 2, 3, 4 and 5. All sequence data generated for this study can be accessed via GenBank: https://www.ncbi.nlm.nih.gov/genbank/. Alignments are available at TreeBase (ID: 26272).

## Abbreviations

BI: Bayesian inference
BPP: Bayesian posterior probability
CB: Cotton Blue
CTAB: Cetyl-trimethyl-ammonium bromide
IKI: Melzer’s reagent
ITS: Nuclear ribosomal internal transcribed spacer
KOH: 5% potassium hydroxide
ML: Maximum likelihood
mPTP: Multi-rate Poisson Tree Processes
PCR: Polymerase chain reaction
RTL: RefSeq Targeted Loci
