# Supplementary figures and images for: Addressing widespread misidentifications of traditional medicinal mushrooms in Sanghuangporus (Basidiomycota) through ITS barcoding and designation of reference sequences
Source: IMA Fungus. 2021 Apr 15;12:10. doi: 10.1186/s43008-021-00059-x (PMC8048060; doi:10.1186/s43008-021-00059-x)

0.0177702

Sanghuangporus\_microcystideus\_C915609\_KP030787

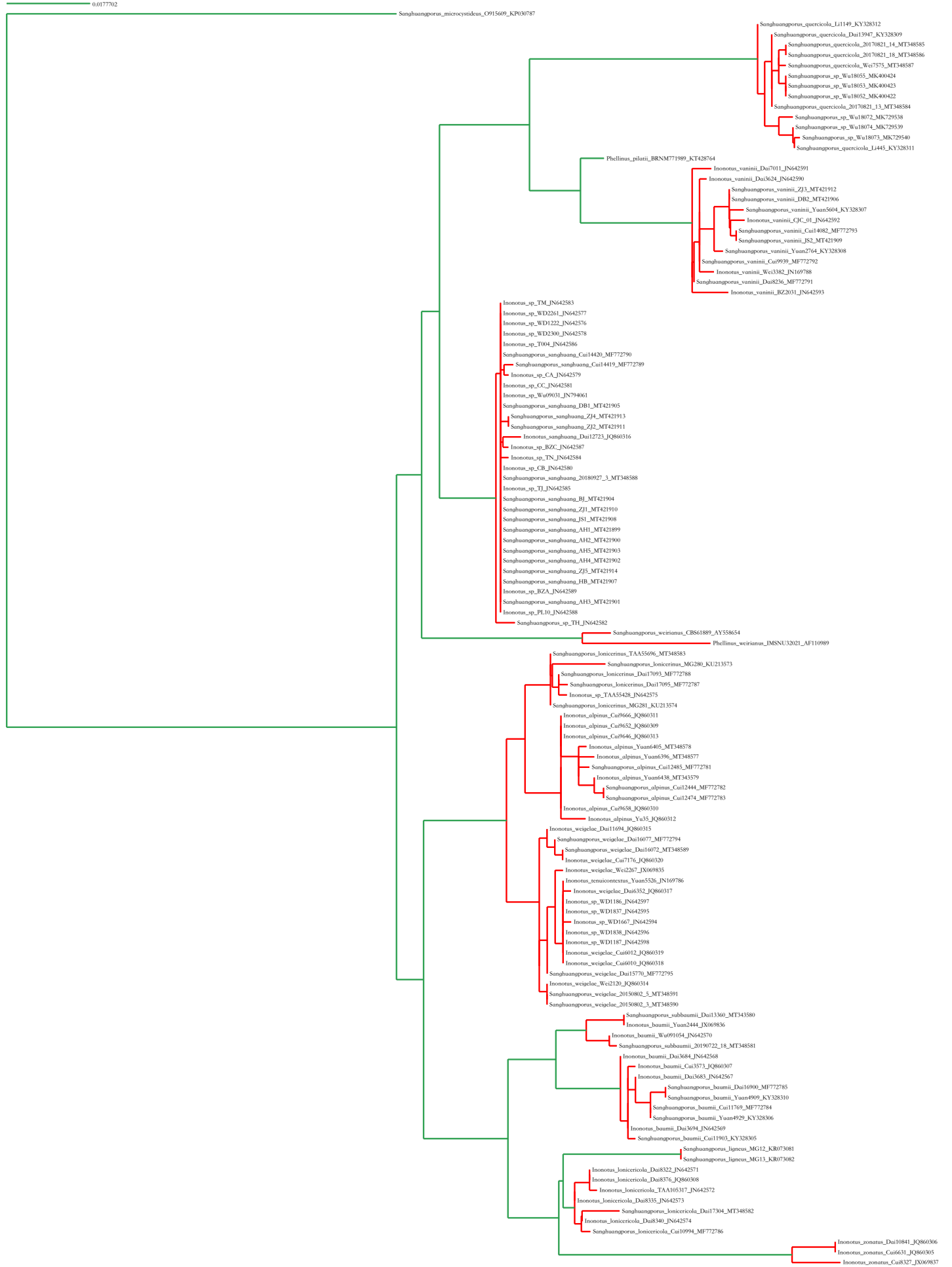

Supplement: Supplementary file 3 — Additional file 3: Figure S1. Molecular species delimitation estimated from the Newick tree file of Fig. 2 using multi-rate Poisson Tree Processes method. The continuous red branches represent a single species. [file 43008_2021_59_MOESM3_ESM.pdf]

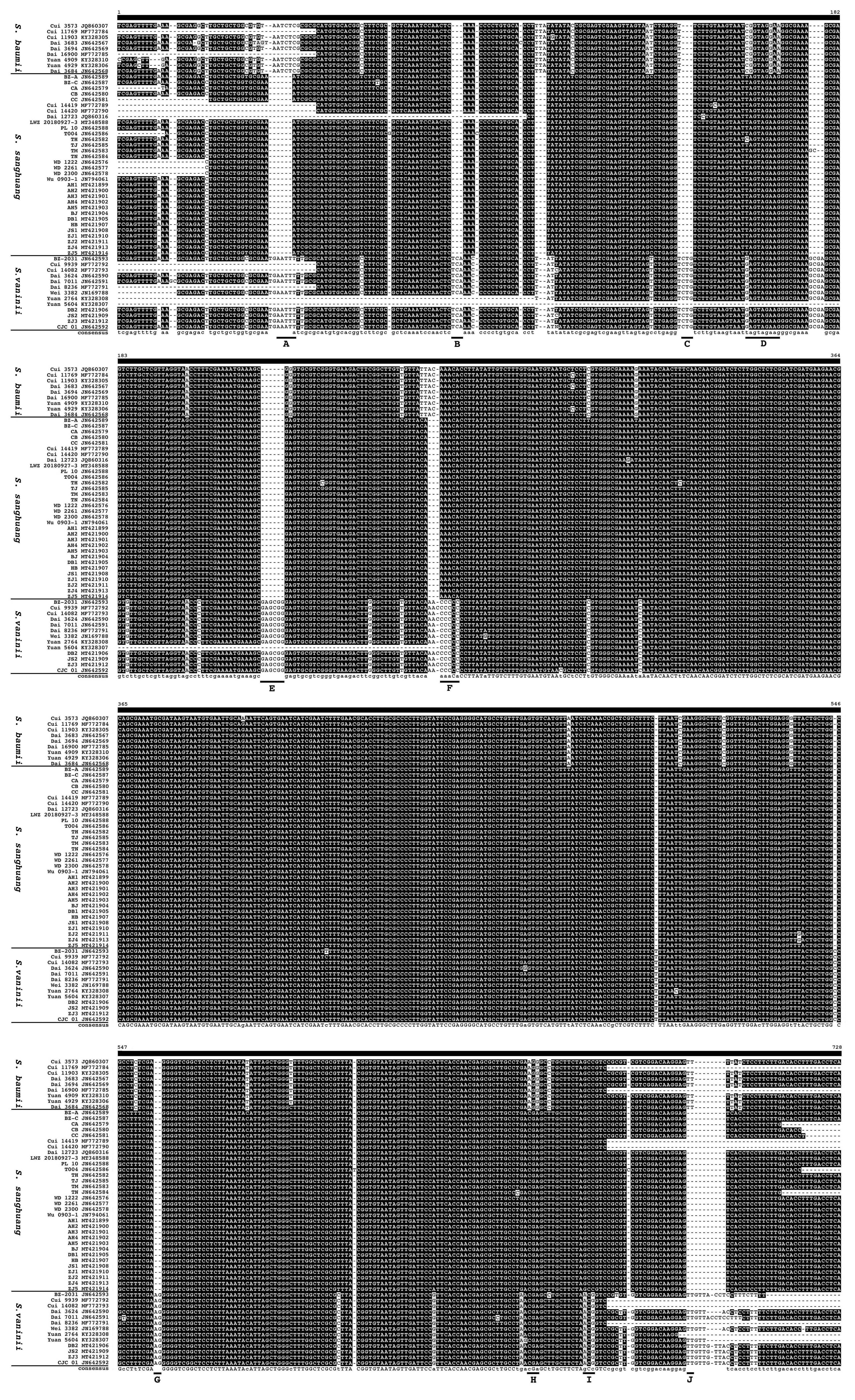

Supplement: Supplementary file 5 — Additional file 5: Figure S2. The alignment of Sanghuangporus baumii, S. sanghuang and S. vaninii generated from ITS sequences submitted by taxonomists. Ten potential diagnostic sequences for Hyperbranched Rolling Circle Amplification are labeled in capital letters. [file 43008_2021_59_MOESM5_ESM.jpg]
