# Supplementary material for: Addressing widespread misidentifications of traditional medicinal mushrooms in Sanghuangporus (Basidiomycota) through ITS barcoding and designation of reference sequences
Source: IMA Fungus. 2021 Apr 15;12:10. doi: 10.1186/s43008-021-00059-x (PMC8048060; doi:10.1186/s43008-021-00059-x)
Supplement: Supplementary file 4 — Additional file 4: Table S1. Genetic distances of ITS sequences between and within species of Sanghuangporus. [file 43008_2021_59_MOESM4_ESM.docx]

Additional file 4: Table S1. Genetic distances of ITS sequences between and within species of *Sanghuangporus*

|  | Species | 1 | 2 | 3 | 4 | 5 | 6 | 7 | 8 | 9 | 10 | 11 | 12 | 13 | 14 |
| --- | --- | --- | --- | --- | --- | --- | --- | --- | --- | --- | --- | --- | --- | --- | --- |
| 1 | *S. alpinus* | *0–1.08%* |  |  |  |  |  |  |  |  |  |  |  |  |  |
| 2 | *S. baumii* | 3.74–5.26% | *0–0.58%* |  |  |  |  |  |  |  |  |  |  |  |  |
| 3 | *S. ligneus* | 4.78–5.57% | 3.56–5.13% | *0%* |  |  |  |  |  |  |  |  |  |  |  |
| 4 | *S. lonicericola* | 3.40–5.11% | 2.19–4.08% | 2.35–2.67% | *0.14–1.16%* |  |  |  |  |  |  |  |  |  |  |
| 5 | *S. lonicerinus* | 1.03–2.86% | 4.57–5.74% | 5.52–6.80% | 4.33–5.57% | *0–1.18%* |  |  |  |  |  |  |  |  |  |
| 6 | *S. microcystideus* | 10.33–11.23% | 11.45–12.13% | 11.69–11.76% | 10.88–11.65% | 10.65–11.27% | *n.a.* |  |  |  |  |  |  |  |  |
| 7 | *S. pilatii* | 4.10–5.19% | 4.93–6.34% | 5.30–5.33% | 4.59–5.16% | 4.77–5.51% | 11.91% | *n.a.* |  |  |  |  |  |  |  |
| 8 | *S. quercicola* | 3.75–7.12% | 4.24–7.47% | 3.40–7.39% | 4.74–7.30% | 4.55–8.01% | 8.90–14.04% | 3.43–5.39% | *0–1.11%* |  |  |  |  |  |  |
| 9 | *S. sanghuang* | 3.42–4.81% | 3.92–5.38% | 5.40–6.26% | 4.43–5.61% | 3.58–4.97% | 9.79–10.81% | 2.74–4.01% | 2.74–6.49% | *0– 0.85%* |  |  |  |  |  |
| 10 | *S. subbaumii* | 3.74–5.32% | 1.19–3.07% | 3.99–4.53% | 2.55–3.79% | 4.28–5.93% | 11.03–11.87% | 5.43–5.87% | 4.40–7.47% | 3.95–5.24% | *0.53–1.30%* |  |  |  |  |
| 11 | *S. vaninii* | 4.25–7.13% | 5.74–8.13% | 4.44–6.82% | 5.60–6.93% | 5.49–7.64% | 11.82–13.13% | 2.69–3.46% | 3.71–6.92% | 3.24–6.05% | 5.69–7.62% | *0–1.72%* |  |  |  |
| 12 | *S. weigelae* | 1.35–2.22% | 4.31–5.21% | 4.82–5.24% | 3.86–5.04% | 1.33–2.96% | 10.46–10.91% | 4.61–5.34% | 4.41–7.86% | 3.42–4.58% | 4.30–5.19% | 5.24–7.81% | *0–0.76%* |  |  |
| 13 | *S. weirianus* | 4.97–7.14% | 5.64–7.50% | 6.15–6.47% | 5.46–7.14% | 5.03–8.32% | 12.46–12.95% | 5.02–5.79% | 5.53–8.77% | 2.90–5.44% | 5.94–7.14% | 5.84–7.90% | 4.90–6.85% | *2.68%* |  |
| 14 | *S. zonatus* | 6.41–7.73% | 5.41–6.57% | 6.51–6.99% | 4.58–5.40% | 7.08–8.97% | 13.22–13.53% | 7.92–8.24% | 7.69–9.70% | 7.17–8.15% | 6.53–6.86% | 7.80–9.16% | 6.81–8.02% | 9.08–10.69% | *0–1.71%* |

The ranges of genetic distances between species are shown below the diagonal, and those within species are shown in italic along the diagonal.
